# Integrating Heat Stress into Circular Livestock Production and Life Cycle Assessment: Toward a Heat-Stress Circularity Index

**DOI:** 10.3390/ani16203290

**Published:** 2026-10-19

**Authors:** Vasileios G. Papatsiros, Georgios I. Papakonstantinou, Christos Eliopoulos, Evangelos-Georgios Stampinas, Konstantina Dimoveli, Lampros Fotos, Nikolaos Tsekouras, Dimitrios Arapoglou, Dimitrios Gougoulis, Georgios Michailidis

**Affiliations:** 1 Clinic of Medicine, Faculty of Veterinary Medicine, University of Thessaly, 43100 Karditsa, Greece; geopapak@vet.uth.gr (G.I.P.); kdimoveli@uth.gr (K.D.); lfotos@uth.gr (L.F.); niktsekou@vet.uth.gr (N.T.); dgoug@uth.gr (D.G.); 2 Institute of Technology of Agricultural Products, Hellenic Agricultural Organization-Demeter (HAO-Demeter), 14123 Athens, Greece; christoseliopoulos@hotmail.com (C.E.); dimarap@yahoo.com (D.A.); 3 Laboratory of Anatomy, Histology and Embryology, Faculty of Veterinary Medicine, University of Thessaly, 43100 Karditsa, Greece; evstampinas@uth.gr; 4 Laboratory of Physiology of Reproduction of Farm Animals, Department of Animal Production, School of Agriculture, Aristotle University of Thessaloniki, 54124 Thessaloniki, Greece

**Keywords:** heat stress, livestock, climate change, circular economy, life cycle assessment, composite indicator, animal welfare, resource efficiency, climate resilience, precision livestock farming

## Abstract

Heat stress (HS) constrains livestock production under climate change, impairing health, welfare, productivity and survival. Because fixed resource use is then spread over less output, HS is also a systems-level sustainability problem. This narrative review, based on a targeted search of PubMed, Web of Science and Scopus (2013–2026), proposes a framework linking climate exposure, animal responses, resource flows, circular interventions and Life Cycle Assessment (LCA) across dairy cattle, pigs, poultry and small ruminants, and grades the evidence behind each link. A decomposition of meta-analytic data indicates that HS raises the impact per kg of milk by approximately 3–12% during heat-stress periods, mainly through non-feed burdens. Monte Carlo analysis shows that the direction of this effect is robust, whereas its magnitude depends mainly on the feed share of the total burden. A Heat-Stress Circularity Index (HSCI) is operationalized with explicit metrics, normalization bounds, a geometric aggregation rule and a minimum-welfare gate. A worked example shows that system rankings depend on the aggregation rule, and a pilot application to a commercial Mediterranean pig farm shows that heat-season piglet and weaner mortality triggers the welfare gate despite moderate productivity retention. Evidence is strong for physiological responses but absent for LCA under measured heat stress; five falsifiable hypotheses, with the corresponding validation designs and sample sizes, are therefore proposed.

## Simple Summary

Farm animals such as dairy cows, pigs, poultry and sheep cope poorly with hot weather, which is becoming more frequent due to climate change. Overheated animals eat less, grow more slowly, produce less milk or eggs, drink more water, fall ill more often and die more frequently. Because farms still need feed, water, energy and buildings, every kilogram of product made during a heat wave can carry a larger environmental footprint. This review links hot weather, animal responses, reuse of farm resources and environmental assessment into one framework. It estimates that heat stress may raise the environmental footprint of milk by roughly 3–12% during hot periods, rates how strong the evidence is for each link, and proposes an index to compare farms. Testing the index on a Greek pig farm showed that piglet and weaner deaths in summer were the most important problems to address.

## 1. Introduction

**Figure 1:**
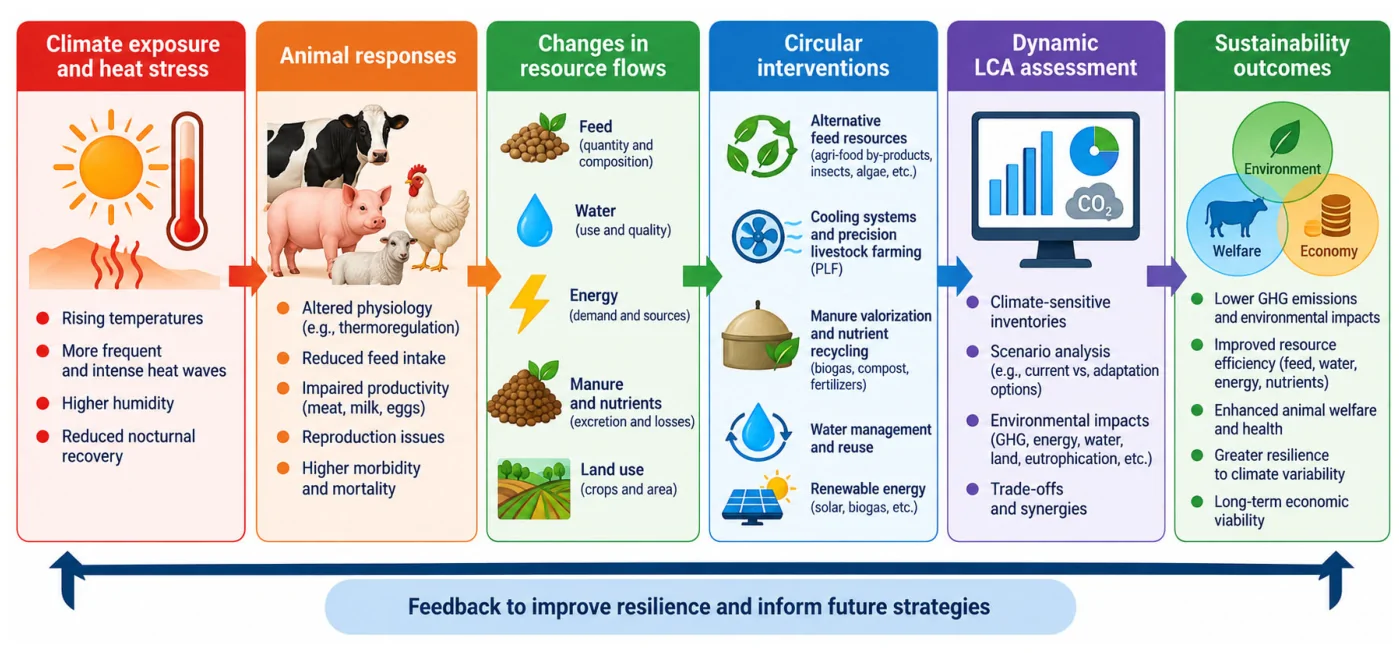
Integrated heat stress–circular economy–life cycle assessment (LCA) pathway for climate-resilient livestock production. Climate exposure and heat stress trigger physiological and productive responses in livestock, which alter feed, water, energy, manure and land-use flows. Circular and adaptation interventions act on these flows, and their net effects are quantified with dynamic, climate-sensitive LCA in terms of environmental, welfare and economic outcomes. The feedback loop indicates that assessment results are used to improve resilience and guide future strategies.

Climate change is progressively altering the environmental conditions under which livestock production takes place [1]. Increasing mean temperatures, more frequent heat waves and changes in humidity and precipitation patterns are expected to increase the exposure of farm animals to thermal stress. Livestock species differ considerably in their thermoregulatory capacity; however, high-producing animals are particularly vulnerable because of their elevated metabolic heat production and comparatively narrow thermal comfort zones [2,3,4]. The overall conceptual relationship among climate exposure, animal response, circular interventions and LCA is summarized in Figure 1.

Heat stress occurs when the animal is unable to dissipate sufficient metabolic and environmental heat to maintain homeothermy. The resulting physiological response includes increased respiratory rate, altered peripheral blood flow, increased water intake, reduced feed intake, changes in endocrine function and redistribution of nutrients away from productive processes [2,5]. In dairy cattle, an increasing temperature–humidity index (THI) is associated with reductions in dry matter intake and milk production, while water intake rises as thermal load increases [6,7]. To illustrate the order of magnitude involved, a recent meta-analysis of 31 studies (34 trials) reported that, across the identified heat-stress trials, dry matter intake (DMI) and energy-corrected milk (ECM) were on average 19.3% and 17.9% lower, respectively, than under thermoneutral conditions, with meta-regression indicating that DMI and ECM decreased by approximately 4.13% and 3.25%, respectively, for each one-unit increase in THI in mid-lactation cows [6]. These pooled dose-response estimates average across studies conducted in different breeds, production systems and climates and should therefore be treated as indicative starting values for the dynamic, climate-sensitive LCA model proposed in Section 10 rather than as fixed, universally applicable coefficients. Effect sizes of this magnitude illustrate why HS-driven productivity losses can be large enough to materially affect impact-per-unit-product calculations, and such dose-response estimates are precisely the type of quantitative input that a dynamic, climate-sensitive LCA model (Section 10) would need to incorporate.

The consequences of HS extend beyond immediate reductions in animal performance. Heat-stressed livestock may experience impaired reproductive efficiency, increased disease susceptibility, altered immune function, poorer welfare and increased culling or mortality risk. These effects can have important economic implications for producers and may increase the environmental burden per unit of animal product when fixed resource requirements are distributed over a lower quantity of output.

This point is particularly important from a life cycle perspective. The environmental impact of livestock production is generally expressed relative to a functional unit such as 1 kg of milk, 1 kg of live weight gain, 1 kg of carcass weight or 1 kg of eggs. When HS reduces productive output without proportionally reducing all upstream inputs, environmental impact per unit of product may increase. Consequently, climate-induced reductions in animal productivity can potentially create an indirect environmental penalty that is not captured by conventional LCA models unless altered production conditions are explicitly incorporated.

At the same time, livestock production is increasingly being considered within the framework of the circular economy. Rather than viewing manure, food-processing residues, agricultural by-products and other materials as waste streams, CE approaches seek to retain their nutritional, energetic and economic value within agricultural systems. Manure, for example, can be transformed into organic fertilizers, biogas and other value-added products, potentially reducing dependence on mineral fertilizers and fossil energy [8]. Similarly, the conversion of food losses and waste into livestock feed has received increasing attention, although environmental performance depends strongly on processing, transportation, allocation and substitution assumptions [9].

Therefore, this review proposes that HS should be conceptualized as a system-level sustainability stressor. The central hypothesis is that climate-induced thermal stress changes animal performance and resource flows, which subsequently modifies the environmental footprint of livestock production. Circular interventions can potentially buffer these effects, but their net sustainability should be assessed using LCA. In quantitative terms, the hypothesis predicts that the ratio of impact intensity under heat stress to that under thermoneutral conditions exceeds 1 whenever the relative loss of output exceeds the feed-weighted relative loss of intake (Section 3.3), a prediction that can be refuted by paired seasonal LCAs within the same farms (Section 15.3).

Beyond synthesizing existing evidence, this review makes five specific contributions: (i) it formalizes the heat-stress impact-intensity mechanism in a simple decomposition and quantifies its plausible magnitude for dairy cows from meta-analytic data, derives its analytical properties and quantifies its uncertainty by Monte Carlo analysis (Section 3.3); (ii) it operationalizes the proposed Heat-Stress Circularity Index (HSCI) by specifying the rationale for each dimension, its metric, normalization bounds, aggregation rule and a minimum-welfare gate, and demonstrates its calculation and sensitivity in a worked example and in a pilot application to a commercial Mediterranean pig farm (Section 13); (iii) it places the Mediterranean evidence in the context of other major heat-exposed production zones (Section 14.2); (iv) it critically grades the strength of the evidence supporting each link of the proposed heat stress–circularity–LCA pathway, distinguishing well-established from inferred relationships (Section 15); and (v) it restates the framework as falsifiable hypotheses, each with a refutation criterion, a study design and, for the central hypothesis, an approximate sample size (Section 15.3).

## 2. Materials and Methods

### 2.1. Review Design and Protocol

This study is conceived as a narrative and exploratory literature review rather than a formal systematic review or meta-analysis. The literature was identified through a targeted keyword search of database metadata (title, abstract and keywords) designed to build a representative thematic corpus rather than an exhaustive systematic compilation; systematic-review reporting conventions were therefore not applied, and the methodology prioritized flexibility and iterative discovery appropriate for mapping a broad, interdisciplinary and rapidly evolving intersection of animal science, environmental assessment and circular economy research. This approach guided the definition of search terms, screening criteria and data extraction procedures used to identify studies addressing heat stress (HS), circular economy (CE) and Life Cycle Assessment (LCA) in livestock production. Unlike a full systematic review, this methodology prioritized an integrative perspective, enabling the inclusion of primary research, systematic reviews, meta-analyses and authoritative institutional reports to capture evidence across diverse technical and disciplinary domains.

### 2.2. Search Strategy and Data Sources

A targeted literature search was conducted across three major academic databases: PubMed, Web of Science and Scopus, covering literature published from January 2013 to August 2026. These databases were selected for their extensive coverage of high-quality, peer-reviewed literature in animal science, veterinary medicine, agricultural engineering and environmental assessment.

The search strategy used combinations of terms grouped into conceptual categories (heat stress concepts, target species/production systems, circularity and LCA concepts), combined using Boolean operators (AND, OR), and applied to the core metadata fields (title, abstract, keywords) of each database’s default search interface.

Core Search String (conceptual): (“heat stress” OR “thermal stress”) AND (“livestock” OR “dairy cattle” OR “beef cattle” OR “pigs” OR “poultry” OR “small ruminants”) AND (“circular economy” OR “circular bioeconomy” OR “life cycle assessment” OR “environmental footprint” OR “water footprint” OR “manure valorization” OR “resource efficiency” OR “climate resilience”)

This conceptual strategy was pragmatically adapted to the syntax and interface of each database without applying complex field codes beyond the default ‘topic’ or ‘all fields’ search, focusing on practical retrieval. Additional search parameters included: publication period 2013–2026; document type restricted to journal articles (primary research, systematic reviews, meta-analyses) and authoritative institutional reports; language restricted to English (Table 1).

Of the 64 included references (Table 1), 14 are primarily focused on circular-economy, life-cycle-assessment or environmental/water-footprint methodology (refs. [8,9,29,43,45,51,54,55,58,59,60,61,62,63]), while the remaining 50 primarily report heat-stress physiology, production, genetics, technology, meat-quality or climate-adaptation evidence in a specific or general livestock context or serve as review-methodology references (e.g., PRISMA 2020 [64]). Five references (refs. [18,19,20,26,57]) were included specifically to broaden the geographical scope of the evidence beyond the Mediterranean region.

### 2.3. Study Selection and Eligibility Criteria

Study selection was conducted using clearly defined eligibility criteria aimed at ensuring thematic relevance and coherence with the review’s objectives, rather than formal methodological appraisal. Titles, abstracts and full texts were screened to identify studies that meaningfully addressed heat stress, circular-economy interventions, or LCA/environmental-footprint assessment in livestock production.

Inclusion Criteria:

- Primary research articles reporting empirical evidence on heat-stress physiology, production impacts, mitigation strategies, or genetic/behavioral adaptation in livestock.
- Studies addressing circular-economy interventions (feed, water, energy, manure/nutrient valorization) relevant to livestock production.
- Studies applying Life Cycle Assessment or related environmental-footprint methodologies to livestock systems.
- Systematic reviews, meta-analyses and authoritative institutional reports synthesizing evidence on the above topics.
- Articles published in peer-reviewed journals or by recognized international institutions (e.g., FAO, IPCC, OECD).

Exclusion Criteria:

- Non-peer-reviewed sources (conference proceedings, theses) other than institutional reports explicitly cited above.
- Studies without original data, without a clearly described mitigation strategy, or without relevance to the HS–CE–LCA intersection.
- Publications addressing genetics, economics, or technology adoption without any connection to heat stress, circularity, or environmental assessment.
- Studies on non-livestock species, unless providing foundational methodological content (e.g., composite-indicator or multi-criteria decision-analysis methods) directly applied within the proposed framework.

### 2.4. Iterative Search and Synthesis Process

To ensure broad coverage of this interdisciplinary field, literature identification followed an iterative and exploratory process. An initial database search provided a core corpus of relevant literature. Subsequently, the reference lists of key studies and seminal reviews were examined to identify additional significant publications—a ‘snowballing’ technique—which was particularly relevant for foundational methodological references falling outside the core search string (e.g., circular-economy definitional work [58], composite-indicator methodology [60], and multi-criteria decision-analysis methods [61], as well as older foundational field and methodological studies from non-Mediterranean heat-exposed regions [19,20,26]). All publications identified through either route were evaluated against the same eligibility criteria stated above. This flexible, discovery-oriented methodology was chosen to construct a nuanced and holistic synthesis over a strictly quantitative and algorithmically reproducible search.

### 2.5. Data Extraction and Thematic Analysis

Data extraction was conducted to capture key descriptive and technical information relevant to the objectives of this narrative review, including study species/system focus, type of heat-stress or circularity evidence, resource-flow or environmental-footprint metrics reported, and proposed or applied mitigation/valorization strategies. Given the heterogeneity of the included studies in terms of species, experimental design, and methodological scope (ranging from primary physiological trials to institutional LCA guidance documents), data extraction was intentionally flexible and adaptive rather than strictly standardized. The collected information was subsequently organized through a thematic analysis approach suitable for synthesizing heterogeneous technological and environmental-assessment literature, supporting an integrative narrative synthesis aimed at identifying recurring themes, quantitative dose-response relationships where available, and knowledge gaps, rather than enabling direct meta-analytic comparison across studies.

### 2.6. Quality Assessment and Synthesis

In line with the narrative synthesis approach, a formal quality-scoring instrument or risk-of-bias tool was not employed. Instead, quality considerations were applied qualitatively to contextualize the robustness, maturity and practical relevance of the included evidence, rather than to rank studies against predefined criteria. For primary research articles and field studies, evaluation focused on the clarity of experimental design, sample size and duration, and transparency of reported effect sizes (e.g., meta-analytic dose-response coefficients versus single-farm observational findings). For institutional reports and methodological references (e.g., FAO, IPCC, OECD documents), consideration was given to their authoritative standing and widespread use as reference frameworks in the LCA and circular-economy literature. The final synthesis integrates findings from primary studies, reviews and institutional guidance in an interpretative and narrative manner, aiming to highlight the strength and quantitative specificity of the underlying evidence—explicitly distinguishing, throughout Section 3, Section 4, Section 5, Section 6, Section 7, Section 8, Section 9, Section 10, Section 11, Section 12, Section 13, Section 14, Section 15 and Section 16, single-study or single-region findings from broader meta-analytic or consensus evidence—rather than to produce a pooled quantitative estimate. In addition, the strength of the evidence supporting each link of the proposed framework was graded on an explicit four-level scale (strong, moderate, limited, absent) according to study design, number of independent studies and geographical breadth; the grading criteria and results are reported in Section 15.1.

### 2.7. Quantitative Analyses

In addition to the narrative synthesis, four original quantitative analyses were performed on published data and are specified here so that they can be reproduced. (i) The analytical properties of the impact-intensity decomposition (Equation (1)), namely its break-even condition and elasticities, were derived in closed form (Section 3.3). (ii) The uncertainty of the decomposition was explored by Monte Carlo simulation (200,000 draws; random seed 2026), with the feed share f drawn from a uniform distribution U(0.4, 0.8) and the pooled relative reductions in dry matter intake (φ) and energy-corrected milk (δ) [6] drawn from uniform distributions spanning ±25% of their point estimates, first independently and then with a correlation of 0.8 between φ and δ imposed through a Gaussian copula. First-order sensitivity indices were estimated as the variance of the conditional mean of the output (50 quantile bins of each input) divided by the total output variance. Because the joint sampling distribution of φ and δ is not reported, the ±25% ranges constitute an assumption-based uncertainty envelope rather than a statistical confidence region. (iii) The robustness of the HSCI to the aggregation rule and weights was assessed by drawing 100,000 weight vectors from a flat Dirichlet (1, 1, 1, 1, 1) distribution (random seed 2026; Section 13.3). (iv) In the pilot application (Section 13.4), the thermal resilience slope was estimated by ordinary least-squares hinge regression of the percentage decline of a composite production index on the THI units above threshold, with 95% confidence intervals based on the t distribution with n − 2 degrees of freedom; its robustness was examined by leave-one-month-out (jackknife) re-estimation, by varying the THI threshold from 70 to 76, by using October alone as the reference period and by fitting the regression through the origin. All analyses were performed in Python 3 (NumPy 2.4.4 and SciPy 1.17.1), and the spreadsheet-compatible calculations are reproduced in Supplementary File S1.

## 3. Heat Stress as a System-Level Challenge in Livestock Production

### 3.1. Biological and Physiological Consequences

The biological response to HS involves a coordinated attempt to maintain core body temperature. Animals initially increase heat dissipation through behavioral and physiological mechanisms, including seeking shade, reducing activity, increasing respiration, increasing water intake and modifying peripheral circulation. When thermal exposure becomes prolonged or severe, compensatory mechanisms may become insufficient and systemic physiological dysfunction can develop [2,4].

THI is one of the most frequently used indicators for evaluating thermal load in livestock, particularly dairy cattle [4]. However, THI alone may not adequately describe the complexity of HS because animal responses depend on breed, age, production level, physiological state, acclimation, air movement, solar radiation and duration of exposure. A climate-sensitive assessment should therefore combine environmental indicators with physiological and behavioral measurements. Complementary indices such as the Black Globe-Humidity Index (BGHI) and the Heat Load Index (HLI), which additionally account for radiant heat load and air movement, may partly address these limitations and merit consideration alongside THI in future climate-sensitive assessments [15,25].

A recent comprehensive review consolidates evidence that heat stress simultaneously compromises production, reproduction and immune competence in dairy cattle, and stresses that physiological indicators (respiration rate, rectal and vaginal temperature) and reproductive endpoints (conception rate, embryo quality) should be interpreted jointly rather than in isolation, since compensatory mechanisms that stabilize one domain (e.g., thermoregulation) frequently come at the expense of another (e.g., fertility or immune function) [10]. This systems-level coupling between physiological and productive responses reinforces the rationale for embedding animal-based indicators directly into circular-economy and LCA frameworks, rather than treating them as a separate welfare add-on.

### 3.2. Heat Stress and Feed Efficiency

Feed represents one of the major environmental inputs of livestock production. Its production requires land, water, energy, fertilizers and other resources and contributes to greenhouse gas emissions. Therefore, changes in feed intake and feed conversion associated with HS can have important implications for environmental sustainability. Reduced feed intake may decrease some absolute emissions, but if productive output declines more rapidly than maintenance-related burdens, the environmental impact per unit of product may increase.

A useful conceptual relationship is: Environmental impact intensity = total life cycle environmental burden/productive output. Under HS, reduced output combined with relatively persistent maintenance requirements can increase impact per unit product. This mechanism may apply to milk, meat and eggs, although its magnitude and direction depend on species, production system and impact category.

This impact-per-unit-product logic primarily reflects an attributional LCA perspective, in which the environmental burden of an animal or herd over a defined period is allocated to a defined functional unit such as 1 kg of milk, meat or eggs. Under this perspective, heat-stress-induced reductions in output can increase impact intensity when upstream inputs do not decline proportionally. However, this relationship should not be interpreted as a universal increase in absolute environmental burden: changes in feeding, herd structure, replacement rates, energy use, mortality and management may alter both the numerator and denominator of the LCA calculation. For intervention and policy questions, a consequential LCA perspective may additionally be required because widespread heat stress can change production decisions, market supply, land use and resource substitution. Accordingly, the framework proposed here treats attributional LCA as the primary approach for farm-level benchmarking, while recognizing consequential LCA as an important extension for regional and sector-level adaptation scenarios.

Heat stress also complicates the greenhouse gas side of the impact-intensity relationship described above. Because absolute enteric methane (CH_4_) production is driven largely by dry matter intake, HS-induced reductions in intake tend to lower total daily CH_4_ output; however, CH_4_ expressed relative to intake (yield) or to milk output (intensity) can increase, since rumen function and milk synthesis are often impaired more than intake itself [11]. In a controlled crossover study in lactating Holstein cows, heat stress reduced absolute daily CH_4_ emissions but increased CH_4_ yield from 14.5 to 17.0 g per kg of dry matter intake and CH_4_ intensity from 9.6 to 11.3 g per kg of energy-corrected milk relative to cooled controls [12]. As this estimate derives from a single controlled crossover trial, it should be regarded as an illustrative, hypothesis-generating value pending confirmation in larger and more diverse cohorts. This pattern illustrates, with a specific greenhouse gas metric, the same structural mechanism proposed for impact intensity more broadly in this review: a fixed or slowly adjusting physiological process (methane-generating rumen fermentation) combined with an HS-driven decline in productive output can raise the environmental burden attributed to each unit of product even when the animal’s absolute emissions fall.

Beef cattle have received comparatively less attention in this review than dairy cattle, reflecting the wider literature base, but the impact-intensity logic developed above applies to beef systems as well, generally with a smaller absolute magnitude. In an early foundational assessment of US livestock industries, heat stress was estimated to cost the beef cow and finishing cattle sectors approximately $369 million annually in aggregate, against $897 million for dairy, reflecting the more extensive, pasture-based nature of most beef cow-calf production and the correspondingly lower cost-effectiveness of heat abatement investment in that sector [21]. At a global scale, a modeling study projected that heat-stress-related losses in milk and meat production from cattle could range from approximately $14.65 billion annually under a low-emissions scenario to $39.94 billion annually under a high-emissions scenario by the end of the century, with disproportionate impacts falling on low- and middle-income countries where cattle represent a larger share of livelihoods and adaptive capacity is more limited [22]. These figures illustrate that the productivity-driven impact-intensity mechanism proposed throughout this review has a substantial and growing economic dimension that complements its environmental one, and that beef systems, despite generally lower unit sensitivity to heat stress than dairy, are far from immune given their global scale.

Field-level evidence from Mediterranean pig production reinforces this multi-level impact-intensity picture. A six-month multilevel field study on a commercial farrow-to-finish farm in Thessaly, Greece, integrating environmental monitoring, continuous Bluetooth Low Energy neck-temperature sensors on lactating sows, oxidative stress biomarkers, a Welfare Quality-based welfare index, and farm production records, found that outdoor THI exceeded the severe-stress threshold (≥90) for over 380 h in July alone, coinciding with a 2.7-fold rise in thiobarbituric acid reactive substances in lactating sows, a reduction in fattening average daily gain from approximately 768 to 610 g per day, and an increase in post-weaning mortality from 5.1% to 18.2% between the coolest and hottest months [27]. This month-to-month contrast reflects observed farm records rather than a formally controlled comparison, and as a single-farm, single-season finding, its magnitude should not be generalized to other operations, regions or years without further replication. Outside the Mediterranean, the same US-wide assessment cited above estimated annual heat-stress losses of approximately $299 million for the swine industry [21], and a multi-model assessment for East Africa found that pigs were exposed to severe heat-stress conditions for more than half of the year across roughly 44% of the region during 1981–2010 [57], indicating that the Thessaly observations are part of a much wider pattern. Histopathological examination of animals that died during the monitoring period revealed lesions consistent with systemic vascular injury, including fibrinous renal microthrombi and splenic lymphocyte depletion, providing a physiological mechanism linking sustained thermal load to the productivity losses and elevated mortality that drive the impact-intensity relationship proposed throughout this review. These findings illustrate, at the level of an individual commercial operation, the same pattern documented at a regional scale for the wider Thessaly plain (Section 14). These species-specific effects on productivity, health and mortality, together with their potential circular-economy and LCA implications, are summarized in Table 2.

Small ruminants deserve additional emphasis given their role in extensive and semi-extensive Mediterranean and dryland systems, where they are frequently the primary livestock species in areas already characterized by high ambient temperatures and limited water availability. Sheep and goats display comparatively greater heat tolerance and lower absolute water requirements than dairy cattle, partly reflecting evolutionary adaptation to arid environments, but heat stress still reduces feed intake, growth and reproductive performance, and grazing behavior is often shifted toward cooler periods of the day, which can reduce effective foraging time and alter pasture-utilization patterns [4,44]. This comparative thermotolerance has been attributed to a combination of morphological and physiological adaptations, including lower relative metabolic heat production and, in some breeds, coat and skin characteristics favoring heat dissipation, although substantial variation exists between breeds and species, with locally adapted breeds generally outperforming high-yielding or introduced genotypes under sustained heat load [39]. Growth performance is nonetheless measurably compromised: reductions in feed intake, average daily gain and feed efficiency have been documented in both sheep and goats subjected to sustained heat load, alongside endocrine changes (altered T3, T4, cortisol, insulin and leptin) that parallel the mechanisms described for cattle and pigs elsewhere in this review [40]. Reproductive function is similarly affected in sheep, with heat stress associated with impaired follicular development, reduced conception rates and altered expression of genes along the hypothalamic-pituitary-gonadal axis [41]. Blood biochemical and energy-metabolite responses to heat stress have also been characterized in indigenous goat breeds, providing physiological benchmarks that could support the development of breed-specific thermotolerance indices for small ruminants, analogous to those discussed for cattle in Section 8 [42]. From a circular-economy perspective, small-ruminant systems are typically less dependent on purchased concentrate feed and mechanical cooling than intensive dairy, pig or poultry systems, which limits the direct energy trade-offs discussed elsewhere in this review but also limits the scope for some circular interventions (e.g., manure-based anaerobic digestion at meaningful scale). Shade provision, rotational grazing timed to avoid peak thermal load, and selection for locally adapted, heat-tolerant breeds are likely to be the most relevant circular and low-input adaptation strategies for these systems, though species-specific quantitative evidence remains considerably sparser than for dairy cattle, pigs and poultry, which is itself identified as a research gap (Section 15). Beyond management-level adaptations, pharmacological antioxidant interventions have also been explored for small ruminants: melatonin implants administered to heat-stressed ewes during summer were associated with better-preserved red blood cell morphology (fewer echinocytes and polychromatophils) and platelet function, alongside a smaller reduction in mean corpuscular hemoglobin concentration relative to untreated heat-stressed controls, consistent with a redox-mediated protective mechanism [38]. Such interventions illustrate a lower-input adaptation option that, unlike mechanical cooling, requires no additional energy or water input, though its net circular-economy and LCA implications, including production costs and disposal of implants, have not yet been evaluated and would need to be established before it could be incorporated into the framework proposed in this review.

### 3.3. Quantifying the Impact-Intensity Mechanism: A Worked Decomposition

The impact-intensity logic described in Section 3.2 can be made explicit with a simple decomposition. Let the total burden of a production system over a heat-stress period be split into a feed-related share *f*, which scales with feed intake, and a non-feed share (1 − *f*) (housing, energy, replacement animals and infrastructure), which is assumed to remain constant over the period. If heat stress reduces feed intake by a proportion φ and productive output by a proportion δ, the ratio of impact intensity under heat stress (I_HS_) to that under thermoneutral conditions (I_TN_) is:

I_HS_/I_TN_ = (1 − *f*·φ)/(1 − δ) (1)

Equation (1) implies that heat stress increases impact per unit of product whenever *f*·φ < δ, i.e., unless intake declines proportionally more than output and feed dominates the total burden. Applying the pooled meta-analytic estimates for dairy cows (φ = 0.193 for DMI and δ = 0.179 for ECM [6]) gives an increase in impact intensity of 12.4%, 10.0%, 7.7%, 5.3% and 3.0% for feed shares of 0.4, 0.5, 0.6, 0.7 and 0.8, respectively. Two implications follow. First, because DMI and ECM decline by similar proportions, the per-kg penalty of heat stress in dairy cows is driven mainly by non-feed burdens; systems with high fixed burdens, including those relying on energy-intensive cooling infrastructure, are therefore proportionally more exposed. Second, the marginal effect of a single additional THI unit (φ = 0.0413, δ = 0.0325 [6]) is small (≤1.7% for *f* ≥ 0.4 and practically nil at *f* = 0.8), so the penalty accumulates with the number of THI units above the threshold and with the number of heat-stress days. Repeated moderate exposure, such as the multi-month exposure documented for lowland Thessaly [52], can therefore generate a larger annual penalty than rare extreme events.

Equation (1) also has closed-form properties that show which parameters govern the penalty. The penalty is zero at the break-even condition δ = f·φ, and the elasticities of the intensity ratio R = I_HS_/I_TN_ with respect to its inputs are:

∂lnR/∂lnδ = δ/(1 − δ);   ∂lnR/∂lnφ = ∂lnR/∂lnf = −f·φ/(1 − f·φ)(2)

At the pooled dairy estimates and f = 0.6, the elasticities are 0.22 for δ and −0.13 for φ and f; a 10% relative error in the estimated loss of output therefore changes R by approximately 2.2%, whereas the same relative error in the loss of intake or in the feed share changes it by approximately 1.3%. Because both elasticities increase with the size of the losses, the uncertainty of the penalty is greatest under severe heat stress.

To quantify the uncertainty of the 3–12% range, a Monte Carlo global sensitivity analysis was performed (Section 2.7). With f drawn from U(0.4, 0.8) and φ and δ varying independently by ±25% around the pooled values [6], the median increase in impact intensity was 7.8% (95% interval −1.6 to 17.0%), and an increase occurred in 94.5% of simulations. Because reduced intake is a major cause of reduced milk output under heat stress [2,6], φ and δ are expected to be positively correlated; when a correlation of 0.8 was imposed, the 95% interval narrowed to 1.4–14.9% (median 7.6%), and an increase occurred in 99.4% of simulations. Under correlated inputs, the first-order sensitivity indices attributed approximately 60% of the output variance to the feed share f, 26% to δ and 4% to φ (under independence, 50% to δ, 32% to f and 18% to φ). The direction of the heat-stress penalty is therefore robust, whereas its magnitude depends mainly on a farm-specific inventory parameter, the feed share of the total burden, which is not reported in the physiological literature and must be measured within the LCA itself rather than assumed.

Equation (1) is conservative for the climate-change impact category because it assumes that emissions scale with intake. In the only controlled trial available, heat stress increased CH_4_ intensity by approximately 18% (from 9.6 to 11.3 g/kg ECM) [12], which exceeds the feed-scaled estimates above; if confirmed, the climate-change penalty of heat stress would be larger than the penalty for feed- or land-driven impact categories. Increased culling and replacement rates would further enlarge the non-feed term. Annual effects scale approximately with the proportion of annual output produced under heat-stress conditions; for example, if one-third of annual output were produced under the pooled heat-stress conditions of [6], the annual increase in impact intensity would be of the order of 1–4%. These values derive from pooled estimates and simplifying assumptions (constant non-feed burden, unchanged herd structure) and should be regarded as bounding calculations for hypothesis testing rather than as measured LCA results.

## 4. Heat Stress, Water and Energy Requirements

### 4.1. Water

Water is essential for thermoregulation, and animals exposed to high thermal loads increase water intake. The magnitude of this response varies according to species, production level, diet and environmental conditions. Recent meta-analytic evidence in Holstein dairy cows indicates a marked increase in water intake during HS, reinforcing the importance of water availability and water-use efficiency in climate-resilient livestock systems [7]. Across 17 studies, heat-stressed cows drank on average about 10 L/day more water than cows under thermoneutral conditions, and water intake rose by approximately 1.5 L per unit increase in THI, although between-study heterogeneity was substantial [7].

At a global scale, the water footprint associated with animal products is already substantially larger, per unit of protein or calorie, than that of most crop products, reflecting the combined blue, green and grey water embedded in feed production, drinking water and processing [62]. Because heat stress increases drinking-water intake specifically at the animal level, its effect should be understood as an incremental addition to an already water-intensive baseline rather than as a separate, isolated burden. This distinction matters when prioritizing where circular water interventions (Section 5) can deliver the largest proportional benefit.

From a CE and LCA perspective, greater water consumption increases direct farm water demand and can also affect pumping, treatment, storage and wastewater management. Potential interventions include rainwater harvesting, reduction of leakage, optimized drinking-point design, monitoring of water intake and carefully controlled reuse of suitable water streams. Water reuse must remain subject to veterinary, microbiological and food-safety requirements.

Quantifying the additional water burden imposed by heat stress is not straightforward, because water intake responds non-linearly to thermal load and interacts with diet composition, milk yield and evaporative losses from cooling systems (e.g., sprinklers, misting). Where evaporative cooling is used to alleviate heat stress, the water consumed for cooling should be added to drinking-water intake within the LCA water-footprint boundary; failure to do so risks systematically underestimating the blue-water footprint of heat-adapted production systems, particularly in water-scarce regions where this omission has the largest practical consequences.

Beyond quantifying volumetric water use, evaluating the environmental significance of heat-stress-driven water demand requires characterizing where and when that water is withdrawn. The AWARE method [63] can help represent water-scarcity impacts, consistent with FAO LEAP guidance on assessing water use in livestock production systems [55], while farm-level assessments should distinguish drinking water from water used for cooling, cleaning and feed production. This distinction is particularly important in hot regions where peak livestock water demand may coincide with seasonal water scarcity.

### 4.2. Energy

Heat mitigation frequently requires additional energy. Fans, evaporative cooling systems, pumps, ventilation and other technologies may increase electricity consumption during periods of high thermal load. This creates a potential trade-off: heat stress increases cooling requirements, which can increase electricity use and associated greenhouse gas emissions. Renewable energy can reduce this burden, but the manufacture, replacement and disposal of energy infrastructure should also be included in LCA where relevant.

The magnitude of this energy burden, and its position within the overall life cycle, can be substantial. A recent life cycle assessment of dairy barns that combined building information modeling with dynamic energy simulation found that operational energy use, dominated by ventilation and cooling, accounted for 57 to 74% of total life cycle carbon emissions across two barn designs, exceeding the embodied emissions from construction materials even though the latter are incurred only once per building lifetime [17]. This finding illustrates why cooling-related electricity demand, rather than the manufacture of cooling equipment itself, is likely to be the dominant driver of the climate impact of heat-abatement infrastructure over a barn’s operating life, reinforcing the need for the operational-energy accounting emphasized throughout this review.

Renewable-powered cooling systems illustrate that this energy trade-off is not automatically resolved simply by substituting fossil electricity with an on-site renewable source. A complete assessment should include the life cycle burdens of photovoltaic or other renewable-energy infrastructure, storage, cooling equipment and replacement components, together with operational energy savings. The net benefit is therefore system- and context-dependent.

## 5. Circular Economy in Livestock Production

The circular economy (CE) concept, for which more than 100 definitions exist in the literature [58], is used throughout this review to describe strategies that retain the value of biological materials, nutrients, water and energy within production systems and reduce dependence on virgin resources. CE should not, however, be treated as synonymous with every heat-stress mitigation strategy. Accordingly, the manuscript distinguishes (i) circular-resource interventions, such as manure nutrient recovery, by-product feed, water reuse and renewable-energy integration; (ii) resource-efficiency interventions, such as precision cooling and sensor-based control; and (iii) climate-adaptation interventions, such as shade, housing modification and heat-resilient genetic selection. This distinction is important because an intervention may improve climate resilience or animal welfare without necessarily creating a circular resource flow. The main circular resource streams relevant to heat-stress adaptation, together with their conventional versus circular management approaches, are outlined in Table 3, together with the corresponding mitigation strategies.

### 5.1. From Linear to Circular Livestock Systems

**Figure 2:**
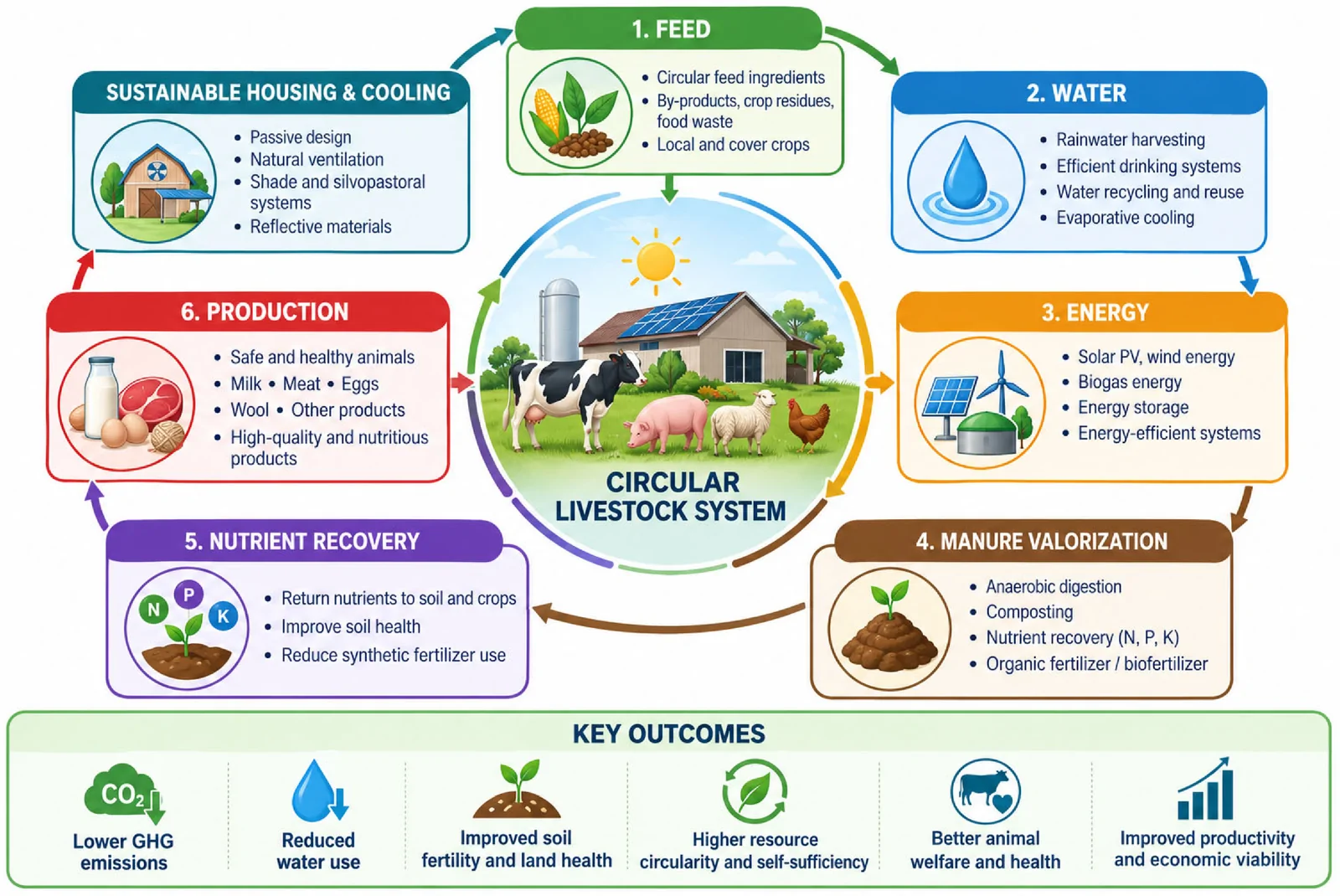
Circular pathway for climate-resilient livestock production. Circular feed resources, water management and reuse, renewable energy, manure valorization and nutrient recovery are linked in a closed loop with animal production and with sustainable housing and cooling. The outcomes shown (lower greenhouse gas emissions, reduced water use, improved soil fertility, higher resource circularity, better animal welfare and improved productivity) are potential benefits whose magnitude depends on local conditions and should be verified by LCA.

The circular resource pathway proposed for climate-resilient livestock systems is illustrated in Figure 2.

The objective is to retain nutrients, energy, water and materials at their highest practical value for as long as possible. FAO has emphasized the role of livestock in circular bioeconomy systems through nutrient recycling, conversion of non-edible biomass into food, and recovery of energy and organic fertilizer from animal residues [45].

### 5.2. Circular Feed Resources

Feed production is often one of the largest contributors to the environmental footprint of livestock products. Substituting conventional feed ingredients with appropriately processed agricultural or food-industry by-products may improve circularity and reduce pressure on land and other resources. Nevertheless, environmental benefits cannot be assumed automatically. Goldáraz-Salamero et al. [9] showed that LCA results for food-loss-and-waste-to-feed systems depend strongly on processing, transport, allocation and substitution assumptions. Circular feed strategies should therefore be assessed for nutritional quality, animal health, productivity, logistics and life cycle performance simultaneously.

A broader systematic review of agri-food LCA reaches a similar conclusion and identifies inconsistent functional units, system boundaries and allocation rules as a persistent problem [59]; circular feed interventions should therefore be benchmarked with consistent functional units (e.g., impact per kg of edible product) under both thermoneutral and heat-stress conditions, and “circular” should not be treated as an automatic proxy for environmentally superior.

## 6. Nutritional Strategies, Circular Feed Resources and Heat-Stress Resilience

Nutritional management represents one of the most flexible strategies for reducing the biological and productive consequences of HS. Thermal stress is associated with reduced feed intake and changes in nutrient partitioning, endocrine function, intestinal integrity and oxidative status. Nutritional interventions therefore have potential to contribute to climate resilience, but their environmental performance must be evaluated across the entire life cycle.

In pigs and poultry, recent reviews describe the effects of HS on nutrient absorption, gastrointestinal integrity, immunity, oxidative balance, growth and reproduction, and discuss nutritional strategies including antioxidants, electrolytes, vitamins, minerals, phytogenic compounds and other functional additives [28,34,36]. Beyond live-animal performance, HS and the acute thermal load experienced during pre-slaughter transport can compromise final product quality in both pigs and poultry, favoring the development of pale, soft and exudative (PSE) meat—a quality defect with direct economic and circular-economy relevance because a lower-value end product effectively increases the environmental impact allocated per unit of usable protein [56].

## 7. Phytogenics and Functional Feed Additives

Plant-derived feed additives may influence antioxidant status, immune function, intestinal integrity and nutrient utilization. However, their sustainability cannot be inferred simply from the fact that they are natural or plant-derived. Land requirements, irrigation, extraction, energy consumption, transport and inclusion rate should be considered in LCA [36].

Field-level support for this pathway comes from a randomized trial in primiparous sows exposed to heat stress during late gestation and early lactation, in which two phenolic phytogenic additives reduced plasma thiobarbituric acid reactive substances by approximately 15% and protein carbonyls by 19 to 23% relative to unsupplemented controls, alongside fewer stillborn piglets and a shorter weaning-to-estrus interval [30]. Because these additives are dosed at only a few grams per animal per day, their direct feed-volume and land-use footprint is small relative to their potential to reduce heat-stress-driven productivity losses, illustrating how a modest circular-economy input (a plant-derived by-product-based additive) may yield a disproportionate reduction in the environmental impact intensity associated with reproductive failure and piglet mortality.

This creates opportunities to connect phytogenic supplementation with agro-industrial circularity when plant residues or processing by-products can be transformed into functional feed ingredients.

## 8. Genetics and Biological Adaptation

Genetic selection for heat tolerance represents a long-term adaptation strategy whose direct environmental burden may be relatively low compared with continuous energy-intensive cooling. The objective is to maintain an acceptable proportion of productive performance under thermal stress rather than maximizing output only under thermoneutral conditions. Global modeling indicates substantial differences in physiological adaptability among livestock species and populations, highlighting the importance of climate-resilient breeding [47].

In poultry, genomic approaches have been proposed to identify biological pathways associated with thermal tolerance and support the development of climate-resilient populations [35]. Genetic resilience should not replace environmental management; rather, it should complement cooling, nutrition and housing adaptation. In pigs, the evidence base for genotype-by-climate interactions is comparatively thinner than in dairy cattle, though it is growing. A systematic review spanning cattle, pigs and chickens found that genotype-by-climate interaction studies exist for all three groups but are least numerous and least experimentally rigorous for pigs. Essentially, all chicken-focused studies to date have been controlled experiments rather than field observations, in contrast to the mostly observational, farm-based evidence available for cattle [48].

A recurring finding in the genetics literature is an unfavorable genetic correlation between production traits and heat tolerance: selection focused narrowly on yield can gradually erode thermotolerance, so combined selection indices that explicitly weight resilience alongside productivity are increasingly recommended [13,23]. We propose that, from a circular-economy and LCA perspective, this trade-off matters because a herd with eroded thermotolerance may require progressively more energy-intensive cooling to sustain output, effectively substituting a recurring operational (energy) burden for a one-time genetic investment. Emerging tools such as marker-assisted selection, genomic breeding values for heat tolerance, and prospective gene-editing or introgression strategies (e.g., the “slick” hair-coat variant in dairy cattle) offer pathways to decouple production from thermal sensitivity, but their environmental footprint, regulatory status and long-term consequences for genetic diversity have not yet been assessed within an LCA framework and should be considered a priority research gap [24].

## 9. Precision Livestock Farming and Artificial Intelligence

**Figure 3:**
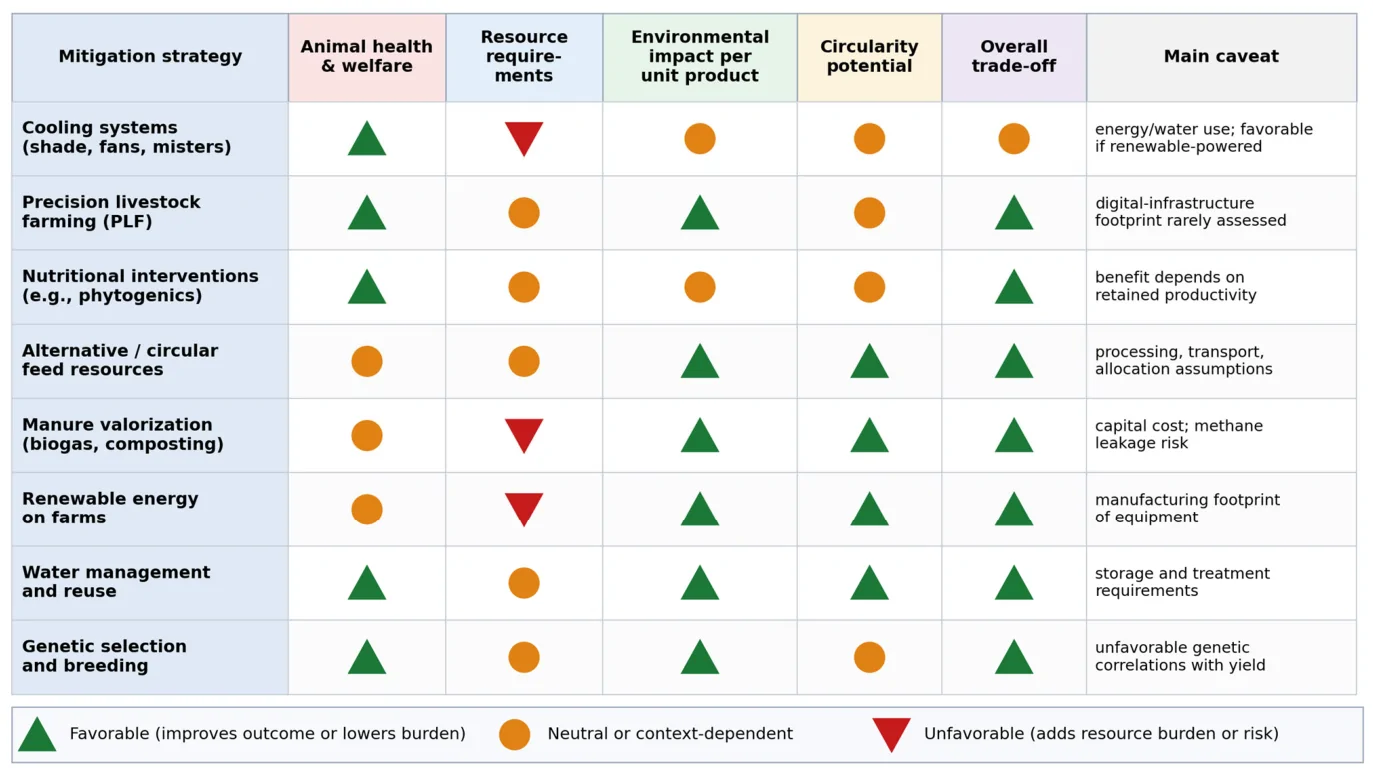
Trade-offs of heat-stress mitigation strategies from a life cycle assessment (LCA) perspective. Eight mitigation and adaptation strategies are rated for animal health and welfare, resource requirements, environmental impact per unit of product, circularity potential, overall trade-off, and the main caveat of each strategy is indicated. Upward triangles, circles and downward triangles denote favorable, neutral or context-dependent, and unfavorable effects, respectively. Ratings are qualitative, literature-informed and consistent with Table 3; environmental impact is judged per unit of product relative to the same heat-stressed system without the strategy. The direction and magnitude of effects should be evaluated within the boundaries of the specific production system.

Precision livestock farming provides an important bridge between animal welfare, resource efficiency and circularity. Beyond stand-alone use, PLF phenotypes are increasingly proposed as an input to genomic selection: high-resolution, continuously recorded indicators such as respiration rate, body temperature and activity can sharpen the accuracy of genomic predictions for thermotolerance relative to conventional pedigree-based breeding values, so that real-time monitoring and long-term genetic selection function as complementary rather than competing strategies [14]. From a circularity perspective, this combined PLF-genomics pathway is attractive because it targets the underlying causes of resource-intensive heat abatement (persistent thermal susceptibility) rather than only its symptoms, potentially reducing the long-run energy and infrastructure burden associated with mechanical cooling. Feeder records and indoor dataloggers already collected on commercial farms can support genuinely predictive heat-stress models without additional hardware, provided that the outcome is modeled at daily resolution and validated with grouped cross-validation [31] (Section 13.4). The principal trade-offs among mitigation effectiveness, additional inputs and environmental performance are summarized in Figure 3.

The sustainability relevance lies in the possibility of replacing continuous resource use with targeted intervention. Continuous cooling may consume electricity even when the biological risk is low, whereas sensor-based systems can activate ventilation or cooling when environmental and physiological indicators exceed thresholds. Thus, digitalization may indirectly reduce environmental impacts by improving the timing and precision of resource use [46].

Recent reviews document a rapidly expanding set of artificial intelligence applications for heat-stress management, spanning behavioral monitoring (feeding, drinking, lying and shade-seeking patterns), physiological prediction (respiration rate, rectal and vaginal temperature from infrared thermography and wearable sensors), and even genomic evaluation of thermotolerance using machine-learning algorithms capable of handling large, non-linear datasets [49]. From an LCA standpoint, however, the environmental cost of the underlying digital infrastructure—sensor manufacturing, data transmission, cloud computing and hardware replacement—is rarely reported alongside the claimed efficiency gains, and closing this gap is essential before precision livestock farming can be credibly labeled as a net-positive circular intervention.

## 10. Dynamic Life Cycle Assessment Under Heat-Stress Conditions

**Figure 4:**
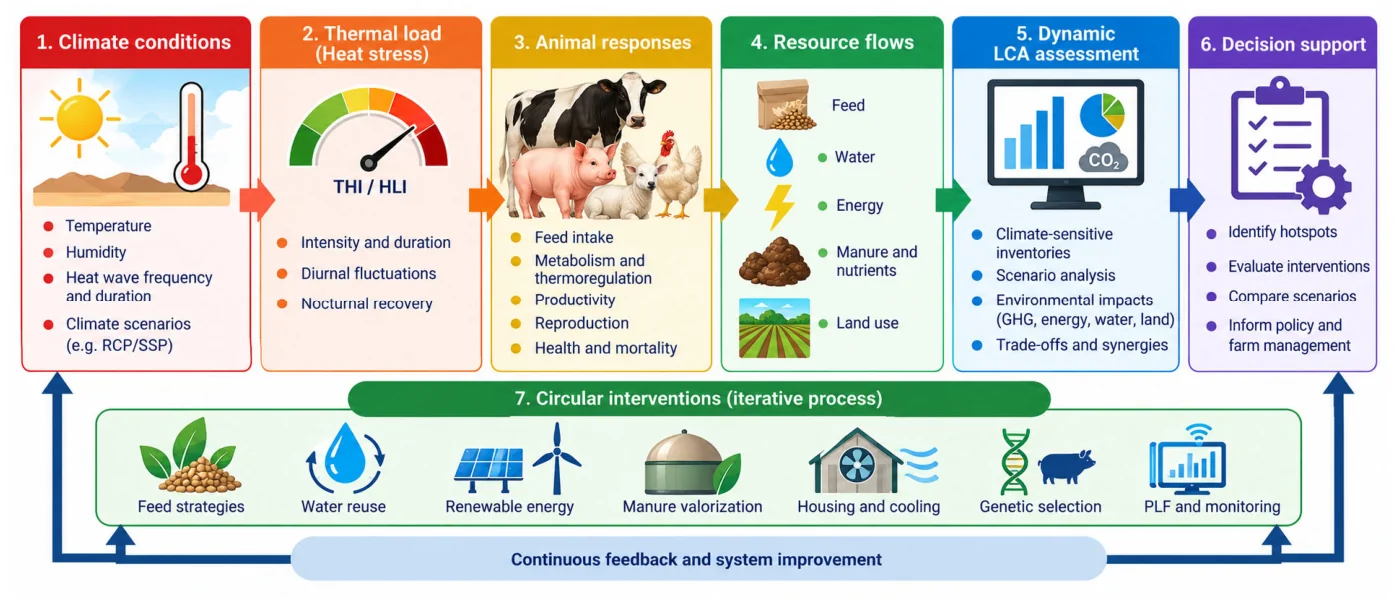
Dynamic climate-sensitive life cycle assessment (LCA) model for heat-stressed livestock production. Climate conditions, including future climate scenarios (**1**), determine the thermal load, expressed by indices such as THI or HLI, together with its intensity, duration and diurnal pattern (**2**). Thermal load drives animal responses in feed intake, thermoregulation, productivity, reproduction, health and mortality (**3**), which alter feed, water, energy, manure and land-use flows (**4**). These flows enter climate-sensitive LCA inventories to quantify environmental impacts, trade-offs and synergies under alternative scenarios (**5**), supporting the identification of hotspots, the evaluation of interventions and farm- and policy-level decisions (**6**). Circular and adaptation interventions (**7**) are introduced iteratively, and continuous feedback improves the system over time.

Conventional LCA generally uses average production parameters. This is a major limitation when evaluating climate-related stress. Climate change is characterized not only by higher annual mean temperature but also by greater heat-wave frequency, longer duration, higher night-time temperatures and altered humidity. A future LCA model should therefore distinguish between thermoneutral and heat-stress periods and quantify changes in feed intake, water consumption, production, mortality, cooling energy, manure and emissions. A dynamic climate-sensitive LCA pathway is presented in Figure 4.

The proposed dynamic framework is climate scenario → environmental exposure → heat-stress intensity and duration → animal physiological response → productivity and health consequences → changes in feed, water and energy requirements → circular interventions → life cycle environmental impacts → net sustainability outcome.

This approach treats the animal as a dynamic biological component of the life cycle model rather than a static production unit. It can therefore capture the environmental consequences of short-term thermal events that annual averages may otherwise hide.

Operationalizing this dynamic approach requires linking downscaled climate projections or observed meteorological data (temperature, humidity, solar radiation, air movement and heat-wave frequency/duration) to species-, breed-, production-stage- and management-specific dose-response functions describing changes in feed intake, water intake, milk or growth performance, reproduction, morbidity and mortality. These response functions should then modify the LCA inventory at an appropriate temporal resolution, preferably daily and, where data permit, hourly during acute heat events. The model should explicitly represent additional cooling electricity and water, changes in feed composition or intake, altered manure outputs, replacement animals and productivity losses. Uncertainty in climate projections, dose-response coefficients and LCA inventory parameters should be propagated through scenario and sensitivity analyses. The appropriate functional unit should also be reported explicitly because heat stress can affect both total farm burden and impact intensity per unit of product in different directions. Equation (1) in Section 3.3 represents the simplest, static form of such an inventory modification, and the pilot application in Section 13.4 shows how the required response functions can be approximated from routine farm records.

### Decision Context and LCA Perspective

The intended decision context should determine the LCA perspective and system boundary. Attributional LCA is most suitable for farm-level benchmarking and comparison of management options within a defined production system. Consequential LCA becomes more relevant when assessing sector-wide adaptation, market responses or large-scale substitution of resources. Intervention assessments should report both the direct environmental effect of the intervention and relevant indirect effects, including the manufacturing and end-of-life burdens of equipment, infrastructure and renewable energy technologies. This distinction is essential because an intervention that appears favorable at the farm level may have different implications when scaled across a region or production sector.

## 11. Climate Scenarios and Livestock LCA

Future climate-sensitive LCA should use multiple scenarios. A low thermal-stress scenario may represent current or modestly changed conditions; moderate and severe scenarios can represent increasingly frequent and prolonged heat waves; and an adapted circular scenario can represent integrated interventions (Table 4). Comparing these scenarios can reveal whether adaptation merely prevents productivity losses or also improves environmental performance.

## 12. Animal Welfare as a Core Component of Sustainable LCA

Environmental LCA alone may classify a system as efficient without adequately considering animal welfare, a limitation increasingly recognized in the broader LCA literature [51]. Heat stress makes this limitation particularly evident. An intervention that reduces emissions but allows animals to remain exposed to severe thermal stress should not automatically be considered sustainable. The proposed framework therefore treats animal welfare as a core sustainability dimension and, for future operational use, recommends that minimum acceptable welfare conditions be treated as constraints rather than as a fully compensatory component of an aggregate score. Environmental and circularity gains should not be allowed to offset unacceptable levels of thermal burden, morbidity or mortality.

Environmental indicators include GHG emissions, water use, land use, eutrophication and energy use. Animal indicators include thermal comfort, morbidity, mortality and reproductive performance. Circularity indicators include nutrient recovery, feed circularity, water reuse and renewable energy share.

A practical implication of combining welfare and environmental indicators is that apparent efficiency gains can mask welfare costs, and vice versa. For example, a system that reduces emissions per unit of product by maintaining animals at the upper limit of their thermal tolerance range may show favorable LCA metrics while systematically compromising comfort and long-term resilience; conversely, an intervention that prioritizes welfare (e.g., aggressive mechanical cooling) may generate a disproportionate energy burden. Reporting welfare and environmental indicators side by side, rather than aggregating them into a single score prematurely, allows decision-makers to identify and discuss such trade-offs explicitly rather than resolving them implicitly through indicator design.

### Economic Sustainability and Life Cycle Costing

Economic sustainability is an important but comparatively underdeveloped component of the proposed framework. Heat stress can generate costs through reduced milk, meat or egg output, impaired reproduction, increased mortality and culling, additional feed and water requirements, and expenditure on ventilation or cooling. Conversely, circular interventions may require capital investment, maintenance and infrastructure while generating savings through reduced purchased energy, recovered nutrients, by-product feed or water reuse. Future applications of the framework should therefore consider life cycle costing (LCC) alongside environmental LCA, using consistent functional units and time horizons. Cost-effectiveness, net present value, payback period and sensitivity to energy and water prices could be reported for major interventions. Integrating economic and environmental outcomes would improve the practical relevance of the framework for farm-level decision making and adaptation investment.

The candidate indicators for each sustainability dimension, their desired direction and their operational definition within the proposed index are specified in Section 13.2 (Table 5).

## 13. Proposed Heat-Stress Circularity Index

A potentially novel contribution of this review is the proposed conceptual Heat-Stress Circularity Index (HSCI) framework. At the present stage, HSCI should not be interpreted as a validated numerical index; rather, it is a structured proposal for integrating thermal resilience, productivity retention, resource circularity, environmental efficiency, animal welfare and, potentially, economic viability. Its value lies in defining a common architecture that can be tested and refined using farm-level data.

A future formulation could be expressed as HSCI = f(TR, PR, RC, EE, W), where TR, PR, RC, EE and W denote thermal resilience, productivity retention, resource circularity, environmental efficiency and welfare, respectively. However, the functional form, normalization, weighting and aggregation method should be established empirically, drawing on established composite-indicator methodology [60]. A multi-criteria approach, such as the Analytic Hierarchy Process [61], may be preferable to a simple additive index because the dimensions are not necessarily substitutable and welfare may require a minimum threshold. The final formulation should therefore be selected after expert elicitation, sensitivity analysis, stakeholder consultation and validation against independent farm-level datasets. A worked operationalization, including the rationale for the selected dimensions, explicit metrics, normalization bounds, aggregation rule, a step-by-step example and a pilot application to farm data, is provided in Section 13.1, Section 13.2, Section 13.3 and Section 13.4.

To operationalize HSCI, each component should have a clearly defined metric, reference population, normalization procedure and data source. Thermal resilience could be represented by heat-load exposure or the rate of productivity decline per unit increase in THI/HLI; productivity retention by actual versus expected thermoneutral output; resource circularity by the proportion of feed, water, energy and nutrients obtained from recovered, recycled or renewable streams; environmental efficiency by life cycle impacts per unit of edible product; and welfare by validated animal-based indicators such as respiration rate, behavioral changes, morbidity and mortality. Before aggregation, each variable should be normalized to a common scale. Weighting should be justified rather than assumed to be equal, and sensitivity analysis should determine whether conclusions are robust to alternative weights. A minimum-welfare threshold should be considered so that unacceptable welfare outcomes cannot be compensated by high circularity or environmental scores.

### 13.1. Rationale for the Selected Dimensions

The five core dimensions were selected so that each corresponds to a distinct node of the causal pathway described in Figure 1 and Section 10, allowing the index to trace the heat-stress effect from exposure to its sustainability consequences. Thermal resilience (TR) captures the sensitivity of the animal response to a given heat load (exposure → response); productivity retention (PR) captures the realized productive consequence (response → output); resource circularity (RC) captures the extent to which the resource flows mobilized under heat stress are supplied from recovered or renewable streams (output → resource flows and circular interventions); environmental efficiency (EE) captures the resulting life cycle penalty per functional unit (resource flows → LCA outcome); and welfare (W) captures the ethical constraint that must hold at every stage. Three selection criteria were applied: (i) correspondence to a distinct node of the pathway; (ii) measurability from routinely available farm records, sensor data or standard LCA inventories; and (iii) conceptual non-redundancy, in line with composite-indicator practice [60].

Economic viability, the sixth candidate dimension mentioned above, is deliberately kept outside the core index and reported alongside it through life cycle costing (Section “Economic Sustainability and Life Cycle Costing”). Monetary values depend on prices, subsidies and exchange rates that change between years and countries, so their inclusion would make HSCI values non-comparable across regions and over time; moreover, a large part of the economic effect of heat stress is already represented through PR. TR and PR are related but not redundant: TR is a sensitivity parameter (the slope of output decline per unit of heat load), whereas PR is a realized outcome that also depends on how much heat load the farm actually experiences, so two farms with identical TR may differ in PR if one is located in a hotter area. Once multi-farm data become available, pairwise rank correlations among the normalized components should be examined; components with |ρ| > 0.8 should be merged or down-weighted, and principal component analysis should be used to confirm the underlying dimensionality [60].

### 13.2. Metrics, Normalization and Aggregation

Table 5 specifies, for each dimension, a candidate metric and the associated sustainability indicators, its normalization to the 0–1 scale, the provisional reference bounds, the desired direction and the data source. Two design choices deserve comment. First, EE is defined as a within-system relative penalty (heat-stress versus thermoneutral impact intensity of the same farm, Equation (1)) rather than as an absolute footprint, so that systems producing different products with different functional units (e.g., milk versus lamb meat) are compared on the heat-stress-attributable part of their footprint rather than on incommensurable absolute footprints. Second, the reference bounds (b_max_ = 5% per THI unit; ∆_max_ = 30%) are provisional values set just above the upper range of published pooled effect sizes [6]; once reference populations exist, they should be replaced by empirical percentiles (e.g., the 5th and 95th percentiles of the observed distribution) and their influence tested in sensitivity analysis.

For aggregation, a weighted geometric mean is proposed as the default: HSCI = x_1_^w1^ × x_2_^w2^ × … × x_5_^w5^, where x_i_ are the normalized component scores (zero values replaced by 0.01) and the weights w_i_ sum to 1. Unlike the arithmetic mean, the geometric mean is only partially compensatory, so a very low score in one dimension cannot be fully offset by high scores in others, which is consistent with the non-substitutability of the dimensions [60]. Equal weights are used as the reference case, and weights derived by expert elicitation (e.g., the Analytic Hierarchy Process [61]) or sampled at random should be used in sensitivity analysis. A minimum-welfare gate is applied before aggregation: if W < 0.5, or if heat-season mortality exceeds 1.5 times the farm’s own thermoneutral-period mortality, the farm is classified as welfare non-compliant and no composite score is reported. The arithmetic mean is reported only as a sensitivity check.

In formal terms, the default rule and its sensitivity check are:

HSCI_G_ = ∏x_i_^w^_i_, HSCI_A_ = Σw_i_·x_i_, Σw_i_ = 1, w_i_ ≥ 0 (3)

Three properties of Equation (3) justify the choice of the geometric mean. First, HSCI_G_ is bounded (0 < HSCI_G_ ≤ 1) and strictly increasing in every component, so an improvement in any dimension can never lower the index. Second, its elasticity with respect to each component is constant and equal to the corresponding weight (∂lnHSCI_G_/∂lnx_i_ = w_i_), which gives the weights a direct interpretation: with equal weights, a 10% relative improvement in any one component raises the index by approximately 2%. Third, the marginal rate of substitution between components i and j is (w_i_/x_i_)/(w_j_/x_j_) under HSCI_G_ but is constant at w_i_/w_j_ under HSCI_A_; the geometric mean therefore rewards the improvement of the weakest component most, whereas the arithmetic mean allows deficits to be fully compensated. By the inequality of arithmetic and geometric means, HSCI_G_ ≤ HSCI_A_, with equality occurring only when all components are equal, and the gap widens as the dispersion of the components increases. Ranking reversals between the two rules are therefore expected whenever two systems have similar arithmetic means but different component dispersion (Section 13.3).

The mortality criterion of the welfare gate is defined on point estimates for practical screening. When the numbers of animals at risk are available, its precision should be reported as the 95% confidence interval of the heat-season/reference mortality rate ratio, estimated with a Poisson or, in the presence of overdispersion, a negative-binomial model with animal-days at risk as an offset. A farm whose entire interval lies above 1.5 is classified as welfare non-compliant with high confidence, and a farm whose interval includes 1.5 is classified as borderline and flagged for continued monitoring.

### 13.3. Worked Example: Cooled Intensive Dairy Versus Pasture-Based Sheep System

To show how the architecture functions, HSCI was calculated step by step for two stylized systems exposed to the same Mediterranean summer, as documented for lowland Thessaly [52]: System A, an intensive Holstein dairy farm with shade, fans and sprinklers; and System B, a semi-extensive, pasture-based dairy sheep flock of a locally adapted breed with natural shade only. Input values were anchored on published estimates where possible and otherwise assumed (Table 6). For System A, an uncooled herd under the pooled meta-analytic conditions would score PR ≈ 0.82 (ECM decline of 17.9% [6]); cooling was assumed to raise PR to 0.95 and to reduce the output-decline slope to 1.0% per THI unit (versus 3.25% in the pooled data [6]), consistent with the long-established effectiveness of shade and evaporative cooling in hot-arid dairies [19], but at the cost of additional electricity, reflected in an assumed impact-intensity penalty of 8% in view of the dominant role of operational energy in barn life cycle emissions [17]. System B was assumed to rely more on non-human-edible feed and on manure recycling on pasture, but to show a steeper output decline and more heat-season days with elevated respiration rates, reflecting the absence of active cooling.

Three findings emerge from the worked example. First, the ranking of the two systems depends on the aggregation rule: under the arithmetic mean, System A scores higher (0.69 vs. 0.66), whereas under the geometric mean, System B scores higher (0.65 vs. 0.61), because the geometric mean penalizes the low resource circularity of System A (RC = 0.19) instead of allowing it to be offset by high thermal resilience and productivity retention. Second, the ranking is sensitive to the weights: when 100,000 weight vectors were drawn at random from a flat Dirichlet distribution, System A ranked higher in 75% of draws under arithmetic aggregation but in only 46% under geometric aggregation, and the 95% range of the score difference (A − B) included zero under both rules (−0.11 to 0.11 and −0.20 to 0.11, respectively). Third, the component profile is more informative than the composite value: System A achieves thermal resilience and productivity retention through energy- and infrastructure-intensive cooling with limited circularity, whereas System B is more circular but less resilient, pointing to different improvement priorities (renewable energy and by-product feeding in System A; shade, breeding for heat tolerance and grazing management in System B). The practical implication is that HSCI should be reported as a component profile together with a pre-specified aggregation rule and a weight-sensitivity analysis, and that single composite values should not be used to rank farms without such analysis.

The reversal follows directly from the properties of Equation (3): the arithmetic means of the two systems differ by only 0.03 (0.69 vs. 0.66), whereas the coefficient of variation of the five components is almost twice as large for System A (0.38) as for System B (0.20), so the geometric mean penalizes System A more strongly.

### 13.4. Pilot Application to a Commercial Mediterranean Pig Farm

**Figure 5:**
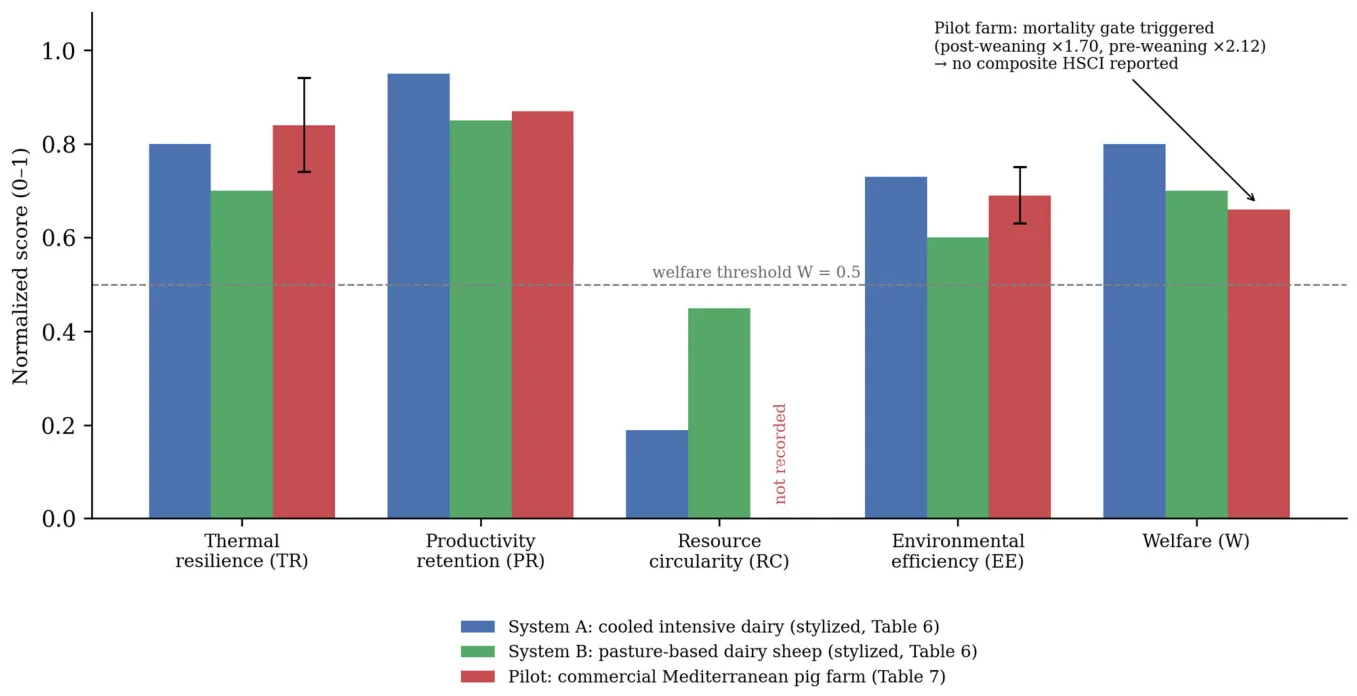
Component profiles of the Heat-Stress Circularity Index (HSCI) for the two stylized systems of the worked example (Table 6) and for the pilot farm (Table 7). Error bars for the pilot farm show the 95% confidence interval of TR and the range of EE across feed shares of 0.4–0.8. The dashed line marks the minimum welfare score; the pilot farm nevertheless fails the welfare gate because of its heat-season mortality ratio, so no composite HSCI is reported for it.

To test whether the index can be computed from data that farms and field studies already produce, HSCI was piloted using the published monthly records of a commercial farrow-to-finish farm in Thessaly (520 sows; natural and mechanical ventilation without active cooling; May–October 2025) [27], supplemented with indoor–outdoor microclimate and lactation feed-intake data from a second, independent commercial farm in the same region and season (a mechanically ventilated farrowing unit with 64 crates) [31]. Because the two data sources refer to different farms, ref. [31] was used only for methodological context and not to compute the components of the pilot farm. June–August (mean outdoor THI 85.3–92.0) were defined as the heat-stress period, and May and October (69.4 and 64.1) as the reference period; because the mean THI in May already exceeded the mild-stress threshold, the reference is conservative and the resulting penalties are, if anything, underestimated. Productive output was represented by a composite production index, calculated as the mean of the ratios of fattening average daily gain (ADG), weaned piglets per sow and farrowing rate to their reference values. The calculations and results are shown in Table 7 and Figure 5 and are reproduced in Supplementary File S1.

Four of the five components could be computed. The composite production index declined by 0.79% per THI unit above the threshold of 72 (95% CI 0.29–1.28; R^2^ = 0.83; n = 6 monthly values), giving TR = 0.84 (95% CI 0.74–0.94), and productivity retention over June–August was PR = 0.87 (ADG 0.82; weaned piglets per sow 0.90; farrowing rate 0.89). A first-order proxy for the environmental penalty, combining the 8.3% longer time to slaughter with the loss of output due to higher post-weaning mortality, gave ∆ = 0.08–0.11 depending on the feed share of total burden (*f* = 0.8–0.4), i.e., EE = 0.63–0.75; because feed conversion was assumed to be unchanged, this is probably an underestimate. W was 0.66 (mean AWI score 13.6 of a possible 40 in June–August across fatteners, weaners and sows, compared with 4.4 in October). Resource circularity could not be computed, because the required data (share of by-product feed, recovered water, renewable energy and recycled manure nutrients) were not recorded in either study.

The point estimate of the TR slope was robust to analytical choices, although its precision was low. Leave-one-month-out (jackknife) estimates ranged from 0.68 to 0.91% per THI unit (TR 0.82–0.86); varying the threshold from 70 to 76 gave b = 0.71–0.99% per THI unit (TR 0.80–0.86); using October alone as the reference period gave b = 0.79%; and a regression constrained through the origin gave b = 0.78% (95% CI 0.52–1.04). The conclusion that the pilot farm retains a moderately high TR is therefore insensitive to these choices, whereas the width of the confidence interval reflects the small number of monthly observations rather than model specification. The precision of the mortality ratios used in the welfare gate could not be estimated, because the monthly numbers of animals at risk were not reported in [27].

The most informative result of the pilot is that the farm fails the minimum-welfare gate: post-weaning mortality in the heat-stress months was 1.70 times, and pre-weaning mortality 2.12 times, that of the reference months (2.43 and 2.64 times, respectively, if October alone is used as the reference). Under the rule defined in Section 13.2, no composite HSCI is therefore reported, irrespective of the missing RC value, because no level of circularity or environmental efficiency may offset this mortality burden. Without the gate, the four available components would have produced a moderately favorable profile (Figure 5), which illustrates why a fully compensatory score would be misleading for this farm. The pilot also identifies the thermal protection of piglets and weaners, rather than further gains in resource efficiency, as the dominant improvement priority.

Three methodological lessons emerge. First, TR estimated from monthly aggregates rests on only four degrees of freedom and has a wide confidence interval; in the farrowing unit, monthly outdoor THI was not correlated with mean lactation feed intake (r = −0.335, *p* = 0.516, n = 6), whereas a model at sow-day resolution based on electronic-feeder records attributed 9.2% of its predictive importance to the building’s cooling offset (∆THI) [31]. TR should therefore be estimated from daily data wherever PLF records exist. Second, month-to-month comparisons are confounded by herd composition: in the farrowing unit, mean batch parity ranged from 2.26 to 3.65 between months, and the month effect on feed intake had to be adjusted for parity [31], so production ratios used for TR and PR should be adjusted for parity or age structure. Third, the provisional bound b_max_ = 5% per THI unit was anchored on dairy data and on a THI formulation different from the one used for pigs [27,31], so species- and index-specific bounds are required before scores are compared across species. Overall, the pilot shows that four of the five HSCI components and the welfare gate can be derived from routinely collected farm records, and that resource-flow data are the main missing element. Neither farm recorded such data; a minimum annual data set for RC would comprise the quantity of by-product or non-human-edible feed, the volume of harvested or recovered water, the renewable share of energy use and the share of manure nitrogen recovered through anaerobic digestion or land application under a nutrient management plan.

## 14. Mediterranean Livestock Systems and Other Heat-Exposed Production Zones

### 14.1. Mediterranean Systems

The proposed framework is particularly relevant to Mediterranean livestock production, where high summer temperatures, heat waves and water scarcity may occur simultaneously. Climate adaptation should not rely exclusively on energy-intensive mechanical cooling. A Mediterranean circular livestock model could combine locally appropriate agro-industrial by-products, solar energy, rainwater harvesting, passive cooling, precision livestock farming, manure valorization and heat-resilient genetics. These interventions should nevertheless be evaluated under local system boundaries because their net benefits depend on climate, water availability, electricity mix, infrastructure requirements, animal species and production system. The framework is developed primarily for Mediterranean and other intensive livestock systems, but the underlying logic may also be adapted to tropical and subtropical smallholder systems, where temperate-tropical crossbred genetics combined with agricultural by-products have similarly been proposed as a heat-stress management strategy for dairy production [16], after appropriate recalibration of species-specific thresholds, resource constraints and circularity indicators.

**Figure 6:**
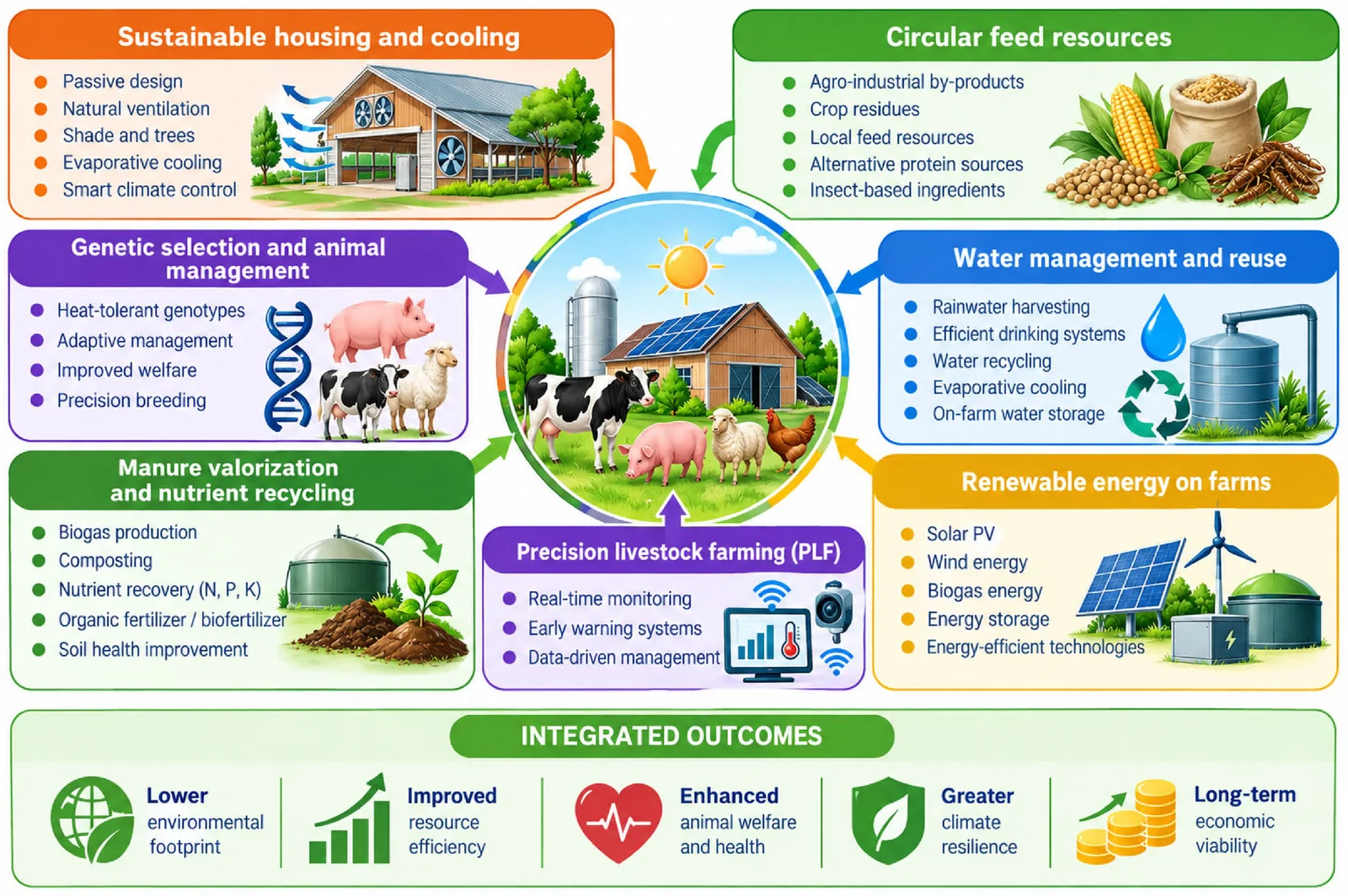
Components of a circular, heat-resilient livestock farm. The integrated framework combines sustainable housing and cooling, circular feed resources, water management and reuse, renewable energy, manure valorization and nutrient recycling, precision livestock farming, and heat-tolerant genetics with adaptive animal management. Together, these components are intended to lower the environmental footprint and to improve resource efficiency, animal welfare and health, climate resilience and long-term economic viability, and they are particularly relevant to Mediterranean production systems exposed to prolonged summer heat stress.

The scale of this exposure is illustrated by a recent spatially explicit assessment of the Thessaly region of Greece, a major Mediterranean livestock-producing area, which used ERA5-Land reanalysis data (2020–2025) to show that outdoor THI regularly exceeded 72 (the threshold associated with productivity decline) across lowland agricultural zones from May through September, reaching 79–82 during peak summer months, with maximum daily air temperatures frequently exceeding 38–40 °C and locally surpassing 45 °C in August. Livestock units in mountainous areas experienced temperatures 6–10 °C lower than those in the lowland plain [52]. These findings indicate that, in intensively farmed Mediterranean lowlands, thermal stress is not confined to isolated heat-wave episodes but constitutes a sustained, multi-month seasonal exposure, reinforcing the case for embedding heat stress as a recurring rather than exceptional condition within circular-economy and LCA models for this region. Figure 6 illustrates the components of such a circular, heat-resilient Mediterranean livestock farm.

### 14.2. Comparison with Other Major Heat-Stressed Production Zones

The Mediterranean evidence reviewed above should be interpreted against the experience of other major heat-exposed production zones, which differ in climate type, production system and dominant adaptation strategy. In the hot-arid dairies of the US Southwest, combinations of shade, fans and evaporative cooling have been developed and evaluated for decades and are regarded as standard practice for maintaining milk yield and reproduction [19]. These systems face the same water–energy trade-off identified here for the Mediterranean because evaporative cooling adds to drinking-water demand in regions where water is scarce. At the national scale, heat stress was estimated to cost US livestock industries approximately $1.7 to $2.4 billion annually, with dairy accounting for the largest share [21]. In feedlot cattle, work in the United States and Australia showed that solar radiation, wind speed and humidity, and not air temperature alone, determine heat load, which led to the development of the Heat Load Index for Australian feedlots [25,26].

Australian dairy systems, which are predominantly pasture-based and therefore exposed to direct solar radiation, provide a leading example of how routine data can be used to quantify thermal resilience: by combining herd-recording test-day data with THI from the nearest weather stations, reaction-norm models were used to estimate the rate of decline in milk, fat and protein yield per unit of THI and to derive genomic breeding values for heat tolerance [18]. This approach corresponds directly to the TR metric proposed for HSCI (Table 5) and shows that this component can be computed at scale from existing data infrastructures. The same work reported unfavorable genetic correlations between heat tolerance and some production traits, consistent with the trade-off discussed in Section 8 [18].

In sub-Saharan Africa, the challenge is primarily one of exposure and adaptive capacity rather than of cooling energy. A multi-model assessment for East Africa showed that severe heat-stress conditions already affected pigs for more than half of the year across roughly 44% of the region during 1981–2010, and projected further increases in the frequency and duration of heat-stress events for several livestock species, including dairy cattle and pigs, with implications for the viability of exotic breeds [57]. Smallholder systems in the region rely on low-input feeding and grazing and have little access to mechanical cooling, and their climate-adaptation research priorities have recently been reviewed systematically [53]. In HSCI terms, such systems are likely to score relatively high for resource circularity but low for thermal resilience and productivity retention, which is the reverse of the profile of cooled intensive systems.

In the humid tropics of Southeast Asia, high humidity reduces the efficiency of evaporative cooling and limits evaporative heat loss by the animal, so temperate high-yielding genotypes are particularly vulnerable. Temperate–tropical crossbreeding combined with locally available agricultural by-products has been proposed as a joint heat-stress and feed-resource strategy for tropical dairy production [16], an approach that aligns closely with the circular-feed and genetic-adaptation pathways of the present framework. Evidence from subtropical environments also indicates that the choice of heat-load indicator matters: in lactating dairy cows in Florida, air temperature alone predicted rectal temperature almost as well as several THI formulations [20], implying that thresholds and index formulas calibrated in one climate should not be transferred uncritically to another.

Taken together, these comparisons indicate that the HSCI components that limit sustainability differ systematically between climates and systems: the water–energy trade-off (RC and EE) dominates in hot-arid intensive systems such as those of the Mediterranean and the US Southwest, whereas thermal resilience and productivity retention (TR and PR) dominate in humid-tropical and smallholder systems. Region-specific reference bounds, and possibly region-specific weights, will therefore be required before HSCI values can be compared across zones.

## 15. Critical Appraisal of the Evidence, Research Gaps and Future Perspectives

### 15.1. Critical Appraisal of the Evidence Base

A critical appraisal of the literature synthesized in this review shows that the evidence supporting the proposed framework is highly uneven across its links (Table 8). Evidence was graded as strong when supported by at least one meta-analysis or by several consistent studies from more than one region; moderate when supported by several primary studies or systematic reviews with some inconsistency in direction or magnitude; limited when resting on a single study, a single farm or a single region; and absent when no direct study was identified. The most important finding of this appraisal is that no study was identified that conducted an LCA of a livestock system under measured heat-stress conditions and compared it with thermoneutral conditions in the same system. The central mechanism of this review, namely that heat stress raises the environmental impact per unit of product, is therefore an inference assembled from separate literatures (physiology, production and LCA methodology) rather than a directly observed result, and it should be treated as a hypothesis to be tested.

The best-supported links concern the physiological and productive responses of dairy cows, for which meta-analyses provide dose-response estimates [6,7]. Even here, three weaknesses limit transferability. First, studies differ in the index and threshold used to define heat stress (different THI formulas and thresholds or alternative indices such as BGHI and HLI [4,15,25]), and in subtropical conditions, air temperature alone predicted rectal temperature almost as well as several THI formulations [20]; pooled slopes are therefore conditional on how exposure was defined. Second, most trials are conducted in climatic chambers over short periods (25 of the 31 studies included in the meta-analysis of [6]), under heat loads that do not reproduce the diurnal cycles, solar radiation and acclimation of field conditions, so their effect sizes may not represent chronic seasonal exposure. Third, the meta-regression slopes refer to mid-lactation cows [6] and cannot be extrapolated to other lactation stages, breeds or species without further data.

The field evidence from Mediterranean pig farms, much of it generated by the authors’ group [27,31], is observational and based on month-to-month comparisons within single farms. Such comparisons are confounded by factors that co-vary with season, including photoperiod, feed batches, pathogen pressure, stocking density and herd age structure, and they lack an unexposed control group; their effect sizes (e.g., the increase in post-weaning mortality from 5.1% to 18.2% [27]) should therefore be regarded as hypothesis-generating rather than as estimates of causal effects. Similarly, the increase in CH_4_ intensity under heat stress rests on a single crossover trial [12], the dominance of operational energy in barn life cycle emissions on a single modeling study of two barn designs [17], and the benefits of phytogenic additives in heat-stressed sows on a single randomized field trial [30].

The circular-economy and LCA literature have a different weakness: its results are sensitive to methodological choices rather than to biological uncertainty. Systematic reviews of food-loss-to-feed systems and of agri-food LCA show that the direction of the environmental benefit can change with allocation, substitution and system-boundary choices [9,59], and claims that precision livestock farming and artificial intelligence reduce resource use are rarely accompanied by an assessment of the footprint of the digital infrastructure itself [46,49,50]. A further concern is that a considerable part of this literature consists of reviews that cite one another, so apparently broad support for a claim may originate from a small number of primary studies. Where the present review relies on such secondary sources, the underlying claims should be regarded as plausible rather than established.

### 15.2. Research Gaps and Future Perspectives

The framework developed in this review is conceptual; translating it into a tool that can genuinely inform livestock management, and environmental policy requires coordinated methodological development. First, heat-stress-sensitive LCA databases and dynamic models should incorporate thermal load, heat-wave duration and species-specific biological response functions rather than relying exclusively on fixed annual production averages. Second, farm-level studies should quantify how discrete heat events alter feed, water, energy, manure and productivity inventories and should propagate these changes into LCA impact categories. Third, both attributional and consequential perspectives should be tested for different decision contexts. Finally, standardized protocols are needed for integrating animal-based welfare indicators with environmental and circularity metrics.

A second cluster of gaps concerns the circular interventions themselves. Renewable-energy-powered cooling, circular feed ingredients, water reuse, manure valorization and nutrient recovery should be evaluated under actual heat-stress conditions rather than assuming that their performance under thermoneutral conditions remains unchanged. Full life cycle system boundaries should include infrastructure, manufacturing, transport, operation and end-of-life where relevant. Comparative studies should also quantify trade-offs, because an intervention that reduces thermal stress may simultaneously increase electricity, water or material requirements.

A third priority concerns animal welfare and genetics. Welfare indicators should be integrated into environmental assessment without allowing environmental efficiency to compensate for unacceptable welfare. Heat resilience should also be considered within breeding and long-term LCA strategies. Species-specific dose-response and LCA data are particularly needed for small ruminants and for production systems outside the regions where current evidence has been generated, such as smallholder livestock systems in sub-Saharan Africa, for which climate-adaptation research priorities have recently been systematically identified [53]. Precision-livestock-farming and genomic-selection approaches should be validated under real farm conditions and assessed for their complete environmental and resource consequences, not only their effects on thermotolerance.

A fourth area concerns economic and technological integration. Economic loss estimates should be linked to environmental impact intensities through combined LCA–LCC or multi-criteria decision frameworks. Such approaches could help identify interventions that are not only biologically effective but also economically feasible and environmentally robust. Artificial intelligence systems that integrate thermal forecasting, sensor-based monitoring and automated feeding, ventilation or cooling control represent another promising area, but their own energy, equipment and data requirements should be included in future sustainability assessments.

Finally, the framework should be tested across contrasting production systems and climates. Mediterranean livestock systems provide an important case because heat exposure may be persistent and seasonally prolonged, but the framework should also be evaluated in tropical, subtropical and arid systems. External validation using independent farm-level datasets is essential before the HSCI or any integrated indicator derived from it is used for benchmarking or policy decisions.

### 15.3. Testable Hypotheses and Design of Validation Studies

To move the framework from a conceptual proposal to a testable scientific model, its central claims are restated below as falsifiable hypotheses, each with the observation that would refute it and the minimum study design required to test it.

H1 (impact intensity). Within the same farm, the attributional impact intensity (e.g., kg CO_2_-eq per kg of fat- and protein-corrected milk or per kg of live weight) is higher in heat-stress periods than in thermoneutral periods (R > 1), and its magnitude agrees with Equation (1) evaluated with farm-specific f, φ and δ. Refutation: R ≤ 1, or systematic disagreement between observed and predicted R. Design: multi-farm study with paired, period-specific LCA inventories within each farm, analyzed with a linear mixed model (period as fixed effect, farm as random effect), with agreement between observed and predicted R assessed by Bland–Altman analysis.

H2 (moderation by the feed share). The heat-stress penalty decreases as the feed share of the total burden increases (Equation (2)). Refutation: no negative association between f and the observed penalty. Design: mixed-model regression or meta-regression of the farm-level penalty on f across farms that differ in housing, energy use and feeding system.

H3 (enteric methane). The increase in CH_4_ intensity under heat stress exceeds the increase predicted from the changes in intake and output alone. Refutation: an increase in CH_4_ intensity equal to or smaller than the feed-scaled prediction. Design: replicated controlled trials (respiration chambers or head-chamber systems) across breeds, lactation stages and diets, extending the single crossover trial currently available [12].

H4 (cooling trade-off). Active cooling lowers impact intensity only if the avoided output loss outweighs the burden that cooling adds. With c denoting the additional burden of cooling as a proportion of the thermoneutral total burden, and φ_c_ and δ_c_ the residual losses of intake and output with cooling, cooling is environmentally beneficial if:

(1 − f·φ_c_ + c)/(1 − δ_c_) < (1 − f·φ)/(1 − δ) (4)

For example, at the pooled uncooled dairy losses [6], f = 0.6 and residual losses of 5% in both intake and output with cooling, Equation (4) is satisfied only if c < 0.053, i.e., if cooling adds less than approximately 5% to the total burden of the heat-stress period. Refutation: interventions that preserve output but raise impact intensity because c exceeds this threshold. Design: controlled on-farm intervention trials with metered cooling electricity and water, analyzed with LCA under both conditions.

H5 (construct validity of the HSCI). The five HSCI components are non-redundant (pairwise |ρ| < 0.8), and HSCI profiles discriminate between farms that differ in heat-abatement and circularity practices. Refutation: strong collinearity among components, or no difference between farms with contrasting practices. Design: cross-sectional multi-farm study with principal component analysis and comparison with independent expert rankings (convergent validity).

For a paired within-farm design, the number of farms required to test H1 can be approximated as:

n = [(z_1−α/2_ + z_1−β_)·σ_d_/∆]^2^ (5)

where ∆ is the mean relative increase in impact intensity to be detected and σ_d_ is the standard deviation of the within-farm differences. No empirical estimate of σ_d_ exists; assuming σ_d_ = 0.10, detecting ∆ = 0.05 with α = 0.05 (two-sided) and 80% power requires 32 farms, and detecting ∆ = 0.08 requires 13 farms; with σ_d_ = 0.15, the corresponding numbers are 71 and 28 farms. These figures are illustrative, but they show that H1 can be tested with a multi-farm study of feasible size, that a single-farm pilot cannot test it, and that the first empirical task is to estimate σ_d_.

## 16. Limitations and Conclusions

### 16.1. Limitations

This review has several limitations. First, it is a targeted narrative rather than a systematic review. Although the databases and thematic search strategy are described, no formal PRISMA screening [64] or quantitative synthesis was undertaken, and selection bias cannot be excluded. Second, a substantial portion of the field-level quantitative evidence discussed throughout the review originates from the authors’ research group and from a limited number of studies in Thessaly, Greece. These studies provide valuable primary evidence but should not be interpreted as externally replicated or representative of livestock systems generally. Comparative evidence from other major heat-exposed production zones has been added (Section 14.2), but the depth of field-level evidence remains greatest for the Mediterranean region. Third, the proposed HSCI and integrated framework have not been empirically validated against independent farm-level or experimental datasets, and its proposed weighting and aggregation approach has been illustrated with a stylized worked example (Section 13.3) and piloted on published records from a single commercial pig farm, in which resource circularity could not be computed (Section 13.4), but it has not been validated against independent multi-farm datasets. The recent Mediterranean studies provide evidence for components of the underlying heat-stress mechanism, but they did not measure HSCI as an integrated construct. Fourth, several proposed dynamic LCA relationships remain theoretical and depend on data that are not yet systematically available in existing LCA databases. Fifth, the evidence base is uneven across species, production systems and geographic regions, with stronger evidence for dairy cattle and pigs than for small ruminants and some extensive systems. Finally, the qualitative classifications of circularity potential and expected LCA effects in the synthesis tables are conceptual and context-dependent; they should not be interpreted as quantitative rankings. In addition, the ranges used in the Monte Carlo analysis of Equation (1) and the variance assumed in the sample-size calculation (Equation (5)) are assumptions rather than empirical estimates and should be updated as farm-level data accumulate. These limitations mean that the framework should currently be used to generate hypotheses and guide research design rather than to make definitive farm-to-farm sustainability comparisons.

### 16.2. Conclusions

Heat stress is not only a challenge for animal health and productivity but also a system-level driver of changes in resource use and environmental impact across livestock production. This review proposes a conceptual framework linking climate exposure, animal responses, resource flows, circular interventions and life cycle assessment, with the proposed Heat-Stress Circularity Index (HSCI) providing a possible architecture for integrating thermal resilience, productivity retention, resource circularity, environmental efficiency and animal welfare. The framework emphasizes that mitigation strategies should be evaluated not only according to their capacity to reduce thermal load or preserve production, but also according to their effects on greenhouse gas emissions, water use, energy demand, nutrient cycling, economic viability and animal welfare. Dynamic, climate-sensitive LCA offers a promising means of representing the temporal effects of heat events, while circular-resource strategies may reduce dependence on virgin inputs when their full life cycle consequences are favorable. Importantly, HSCI remains a conceptual framework rather than a validated metric. Its operationalization will require standardized indicators, species- and system-specific dose-response functions, explicit LCA and LCC boundaries, sensitivity analysis, welfare thresholds and independent farm-level validation. The Mediterranean evidence reviewed here provides a relevant case for testing this approach, but broader validation across climates, species and production systems is necessary before the framework can support benchmarking or policy decisions.

Based on the evidence and analyses presented, five specific conclusions can be drawn. (1) During heat-stress periods, the environmental impact per kg of milk is expected to increase by approximately 3–12% in dairy cows, depending mainly on the share of non-feed burdens (Equation (1)); for the climate-change category, the penalty may be larger because heat stress increased CH_4_ intensity by about 18% in the only available controlled trial. Monte Carlo analysis indicates that the direction of this effect is robust (an increase in 94–99% of simulations), whereas its magnitude depends mainly on the farm-specific feed share of the total burden, which must therefore be measured rather than assumed. (2) The evidence base is asymmetric: the physiological and productive effects of heat stress are well established by meta-analyses, whereas its resource-flow and life cycle consequences rest on single studies or on inference, and no study was identified that performs an LCA under measured heat-stress versus thermoneutral conditions; this is the most important research gap, and a paired within-farm design with approximately 13–71 farms, depending on the effect size and between-farm variability, would be required to test it (Section 15.3). (3) Circular interventions cannot be assumed to be environmentally superior, because their LCA outcomes depend on allocation, substitution and processing assumptions and on their own energy and infrastructure requirements. (4) HSCI can be operationalized with transparent metrics, normalization bounds and a minimum-welfare gate, but the worked example shows that the ranking of contrasting systems reverses with the aggregation rule and weights, and the pilot application to a commercial pig farm shows that the minimum-welfare gate, triggered by heat-season piglet and weaner mortality, can be decisive, while resource-flow data are the main missing input. HSCI should therefore be reported as a component profile with a pre-specified aggregation rule and sensitivity analysis rather than as a single ranking score. (5) The limiting sustainability dimension differs by climate and system—the water–energy trade-off in hot-arid intensive systems such as those of the Mediterranean and the US Southwest, and thermal resilience and productivity retention in humid-tropical and smallholder systems—so adaptation priorities and index reference bounds should be region-specific.

## Figures and Tables

**Table 1:** Distribution of the 64 included references by primary species/production-system focus. This reflects the composition of the final evidence base synthesized in this review and is distinct from raw database search-hit counts. Classification reflects each reference’s predominant focus; references addressing multiple species were assigned to their most prominent focus.

Primary Species/Production-System Focus	Included Refs. (n)	Reference Numbers
Dairy cattle	16	[3,4,5,6,7,10,11,12,13,14,15,16,17,18,19,20]
Beef/general cattle	6	[21,22,23,24,25,26]
Pigs	7	[27,28,29,30,31,32,33]
Poultry	4	[34,35,36,37]
Small ruminants	5	[38,39,40,41,42]
Multi-species/general livestock	18	[2,8,9,43,44,45,46,47,48,49,50,51,52,53,54,55,56,57]
Not species-specific (climate context, CE/LCA methodology and tools)	8	[1,58,59,60,61,62,63,64]

**Table 2:** Heat stress effects on livestock production and potential circular-economy/LCA implications. The LCA and circular-economy implications represent literature-informed conceptual expectations and should not be interpreted as quantitatively validated effects.

Species	Principal HS Effects	Resource-Flow Changes	Potential LCA Consequence	Circular Opportunities	Key Refs.
Dairy cattle	↓ feed intake and milk yield; impaired reproduction; increased water intake	Water ↑; cooling energy ↑; efficiency ↓	Potential ↑ impact/kg milk	Solar cooling; circular feed; manure biogas; water efficiency; precision cooling	[2,3,5,6,7]
Beef cattle	↓ intake and growth; altered thermoregulation; mortality risk	Water ↑; feed efficiency ↓	Potential ↑ impact/kg carcass	Shade; silvopastoral systems; resilient genetics; manure recovery	[3,4]
Pigs	↓ feed intake and growth; reproductive and immune effects	Water ↑; ventilation/cooling energy ↑	Potential ↑ impact/kg live weight	Precision ventilation; circular feed; renewable energy	[28,33,56]
Poultry (broilers)	↓ intake and growth; oxidative and intestinal dysfunction; mortality	Water ↑; ventilation/cooling energy ↑	Potential ↑ impact/kg meat	Smart ventilation; circular feed; renewable cooling	[34,35,37,56]
Poultry (laying hens)	↓ egg production; shell-quality and immune effects	Water ↑; ventilation energy ↑	Potential ↑ impact/egg	Precision cooling; circular feed; renewable energy	[35,36]
Small ruminants (sheep/goats)	↓ intake and growth; reproductive effects; altered grazing	Water ↑; feed availability may be limiting	Context-dependent	Silvopastoral systems; local feed; resilient breeds	[4,39,40,41,42,44]

↑, increase; ↓, decrease. All directions of change are expressed relative to the same species and production system under thermoneutral conditions, evaluated with a standard attributional LCA baseline (impact per functional unit). “Context-dependent” indicates that the direction of change cannot be generalized from the available evidence. Species categories follow those used in Table 1 and in the text (dairy cattle, beef cattle, pigs, poultry, and small ruminants).

**Table 3:** Heat-stress mitigation strategies grouped by resource stream, from a circular economy and LCA perspective. Shaded rows summarize each resource stream (conventional versus circular or adaptive management, relevance under heat stress and potential LCA benefit); the rows below list the corresponding strategies. Qualitative CE and LCA classifications are conceptual, literature-informed and context-dependent rather than validated quantitative scores.

Strategy			Animal Benefit	Resource Requirement	CE Potential	Expected LCA Effect	Trade-Off	Refs.
Heat (thermal load). Conventional: uncontrolled thermal load → circular/adaptive: passive cooling, shade, reflective structures. HS relevance: direct reduction in thermal load. Potential LCA benefit: low-energy adaptation [4].								
Natural shade			High	Low	High	Generally favorable	Land/infrastructure	[4]
Mechanical ventilation			High	Electricity ↑	Moderate	May reduce impact/kg if output preserved	Electricity use	[3,5]
Evaporative cooling			High	Water + electricity ↑	Moderate	Potentially favorable; water footprint may rise	Water use	[3]
Energy. Conventional: grid/fossil electricity → circular: solar PV, biogas energy. HS relevance: cooling increases electricity demand. Potential LCA benefit: lower fossil-energy dependence [45].								
Solar-powered cooling			High	Infrastructure	Very high	Potential reduction in fossil-energy GHGs	Manufacturing footprint	[32,45]
Information. Conventional: periodic observation → circular/efficiency: sensors + AI + PLF. HS relevance: early detection of thermal stress. Potential LCA benefit: reduced unnecessary resource use [46].								
Precision cooling			High	ICT + electricity	Very high	Potential reduction in unnecessary energy	Equipment/data needs	[46]
AI-based prediction	High	ICT infrastructure	Very high	Potential resource optimization			Data/hardware footprint	[46]
Feed. Conventional: conventional/imported ingredients → circular: agro-industrial by-products, food-loss-derived feed. HS relevance: maintains nutrient supply when intake declines. Potential LCA benefit: lower land/feed burden [9,29].								
Circular feed			Moderate–high	Processing/transport dependent	Very high	Potential reduction in land/feed impacts	Nutritional variability	[9,29]
Phytogenic additives			Moderate	Botanical production + processing	Moderate–high	Potentially favorable if productivity improves	Ingredient production burden	[36]
Water. Conventional: freshwater extraction → circular: rainwater harvesting, optimized reuse. HS relevance: water demand rises during HS. Potential LCA benefit: reduced freshwater demand [7].								
Rainwater harvesting			Indirect	Infrastructure	High	Potential reduction in freshwater demand	Storage/treatment	[7]
Manure. Conventional: storage/disposal → circular: anaerobic digestion, compost, nutrient recovery. HS relevance: efficient resource management becomes more important. Potential LCA benefit: energy recovery and fertilizer substitution [8].								
Manure anaerobic digestion			Indirect	Infrastructure + energy	Very high	Energy recovery + manure management	Capital cost; leakage	[8]
Genetic resources. Conventional: selection for maximum production → adaptive: selection for thermal resilience. HS relevance: maintains production under HS. Potential LCA benefit: lower dependence on energy-intensive adaptation [35].								
Heat-resilient genetics	High	Breeding resources	High	Long-term reduction in adaptation inputs			Selection trade-offs	[35]

↑, increase relative to the same heat-stressed system without the strategy. Qualitative descriptors (Low, Moderate, Moderate–high, High, Very high) are ordinal, expert-based ranks of the strategies relative to each other within this table and are not absolute scores. “Expected LCA effect” and “Potential LCA benefit” refer to impact per functional unit relative to the same heat-stressed system without the strategy, both evaluated with a standard attributional LCA; the thermoneutral attributional LCA of the same system is the common reference baseline. Potential LCA benefits depend on system boundaries, allocation, substitution assumptions and local resource conditions.

**Table 4:** Climate-sensitive LCA scenarios for livestock production. The scenario categories are conceptual and intended to guide future quantitative modeling rather than represent measured outcomes.

Scenario	Heat Exposure	Adaptation	Expected Productivity	Resource Demand	Environmental Risk	Refs.
Baseline	Low	Conventional	High	Moderate	Moderate	[43]
Climate change	Moderate	Limited adaptation	↓	Water ↑; energy ↑	High	[1,22]
Severe heat	High	Limited adaptation	↓↓	Water ↑↑; energy ↑	Very high	[22,52]
Conventional adaptation	High	Mechanical cooling	Maintained/↑	Energy ↑↑	Trade-off	[3,5]
Renewable adaptation	High	Solar cooling + PLF	Maintained	Fossil energy ↓	Lower	[45,46]
Circular adaptation	High	Circular feed + water + energy + manure recovery	Maintained	External inputs ↓	Lower	[8,9]
Integrated resilient system	High	CE + LCA + PLF + genetics	High resilience	Optimized	Lowest potential impact	Conceptual framework proposed here

↑/↓, increase/decrease; ↑↑/↓↓, larger expected change. Directions are expressed relative to the baseline scenario, which represents a standard thermoneutral attributional LCA baseline of the same production system. Qualitative descriptors (Low, Moderate, High, Very high) are ordinal and relative among scenarios.

**Table 5:** Operational specification of the Heat-Stress Circularity Index (HSCI) components and associated sustainability indicators for heat-stressed livestock systems. Reference bounds are provisional and should be recalibrated from empirical reference populations; the economic dimension is reported alongside the index and is not aggregated.

Component	Metric and Associated Candidate Indicators	Normalization to 0–1	Provisional Bounds	Desired Direction	Data Source
Thermal resilience (TR)	b: % decline in daily output per THI unit above the species-specific threshold (reaction-norm slope) [6,18]; production retained during heat events	TR = 1 − b/b_max_; truncated to [0, 1]	b_max_ = 5% per THI unit	decreased (↓) b; increased (↑) retained output	Production records and on-farm or nearest weather-station THI [18,31]
Productivity retention (PR)	Y_HS_/Y_TN_: milk, meat or egg output in the heat-stress period divided by the expected thermoneutral output of the same animals; feed efficiency [6]	Ratio, capped at 1	0–1 (natural scale)	↑	Production records; lactation- or growth-curve predictions
Resource circularity (RC)	Mean of four shares: circular feed (dry matter from by-products or non-human-edible biomass) [9]; recovered or harvested water [7]; renewable energy in total energy use, including cooling electricity [45]; manure N/P recovered and recycled [8]	Shares are already on 0–1; arithmetic mean	0–1 (natural scale)	↑	Farm resource inventory
Environmental efficiency (EE)	∆ = I_HS_/I_TN_ − 1: relative increase in impact per functional unit (kg CO_2_-eq/kg product [54]); water use and fossil energy per kg product as additional impact categories in sensitivity analysis	EE = 1 − ∆/∆_max_; ∆ ≤ 0 gives EE = 1; truncated to [0, 1]	∆_max_ = 0.30	↓ ∆	Attributional LCA of heat-stress vs. thermoneutral periods (Equation (1))
Welfare (W)	Proportion of heat-season animal-days with an animal-based indicator (e.g., respiration rate or panting score) above a species-specific threshold; THI exposure; morbidity and mortality [4,5]	W = 1 − proportion	Gate: W ≥ 0.5 and mortality ≤ 1.5 × thermoneutral-period mortality	↓ thermal burden; ↓ mortality	Sensors, visual scoring and mortality records [49,51]
Economic (satellite; not aggregated)	Heat-stress-attributable production loss ($/animal/year); life cycle costing indicators (net present value, payback period) of interventions [21,22]	Not normalized; reported alongside HSCI	—	↓ loss	Farm accounts; LCC (Section “Economic Sustainability and Life Cycle Costing”)

**Table 6:** Worked example of the HSCI calculation for two stylized systems. Values are illustrative; they are partly anchored on published estimates and partly assumed, and do not represent measured farms.

Step/Variable	System A: Cooled Intensive Dairy	System B: Pasture-Based Dairy Sheep	Basis
Output decline slope b (% per THI unit)	1.0	1.5	A: assumed (pooled uncooled value 3.25 [6]); B: assumed
→ TR = 1 − b/5	0.80	0.70	Table 5
PR = Y_HS_/Y_TN_	0.95	0.85	A: assumed with cooling (uncooled 0.82 [6]); B: assumed
Circular feed/water/renewable energy/manure N shares	0.20/0.05/0.10/0.40	0.60/0.20/0.30/0.70	Assumed
→ RC (mean of the four shares)	0.19	0.45	Table 5
Impact-intensity penalty ∆	0.08	0.12	A: cooling electricity [17]; B: Equation (1)-type penalty; both assumed
→ EE = 1 − ∆/0.30	0.73	0.60	Table 5
Proportion of heat-season animal-days above respiration threshold	0.20	0.30	Assumed
→ W = 1 − proportion	0.80	0.70	Welfare gate passed (W ≥ 0.5)
HSCI, arithmetic mean, equal weights	0.69	0.66	Sensitivity check
HSCI, geometric mean, equal weights	0.61	0.65	Default rule
HSCI, geometric mean, welfare weight 0.30 (others 0.175)	0.63	0.65	Sensitivity check
Probability that A scores higher than B over 100,000 random weight vectors (arithmetic/geometric)	0.75/0.46	—	Weights drawn from a flat Dirichlet (1, 1, 1, 1, 1) distribution

**Table 7:** Pilot application of the HSCI to published data from a commercial Mediterranean farrow-to-finish pig farm [27], with contextual data from a separate commercial farrowing unit [31]. Heat-stress period: June–August 2025; reference period: May and October 2025.

Component	Data Used	Calculation	Result	Comment
TR	Monthly outdoor THI and composite production index (ADG, weaned piglets/sow, farrowing rate), May–October [27]	Regression of % decline on THI units above 72; TR = 1 − b/5	b = 0.79% per THI unit (95% CI 0.29–1.28; R^2^ = 0.83); TR = 0.84 (0.74–0.94)	n = 6 monthly values; bound derived from dairy data
PR	Heat-stress vs. reference months [27]	Mean of ratios: ADG 0.82; weaned/sow 0.90; farrowing rate 0.89	PR = 0.87	Reference not strictly thermoneutral (May THI 69.4)
RC	Not recorded	—	Not available	Requires farm resource inventory
EE	Age at slaughter (180.3 vs. 166.5 d) and post-weaning mortality (12.4% vs. 7.3%) [27]	∆ = [(1 − *f*)·D_HS_/D_TN_ + *f*]/S − 1; S = survival ratio; feed conversion assumed unchanged	∆ = 0.08–0.11; EE = 0.63–0.75 (0.69 at *f* = 0.6)	First-order proxy, not an LCA
W	AWI scores, fatteners, weaners and sows [27]	W = 1 − mean AWI (June–August)/40	W = 0.66	AWI 13.6 vs. 4.4 in October
Welfare gate	Pre- and post-weaning mortality [27]	Heat-stress/reference mortality ratio vs. 1.5	Post-weaning 1.70; pre-weaning 2.12: gate triggered	Classified welfare non-compliant; no composite HSCI
Context: building buffering	Indoor vs. outdoor THI, farrowing unit [31]	∆THI = outdoor − indoor THI	∆THI 10.5–14.3; indoor THI 72.4–75.3 (June–August)	Mechanical ventilation only

ADG, average daily gain (fattening); AWI, animal welfare indicator score (0–40, higher = worse); D, age at slaughter; *f*, feed share of total burden; S, (1 − post-weaning mortality in heat-stress months)/(1 − post-weaning mortality in reference months). The two data sources are published field studies; values were extracted from their tables and are reproduced with formulas in Supplementary File S1.

**Table 8:** Strength of evidence supporting the main links of the proposed heat stress–circularity–LCA framework.

Framework Link	Main Evidence	Evidence Type	Strength	Main Limitation
Heat load → feed intake and milk yield (dairy cattle)	[6,10]	Meta-analysis; review	Strong	Heterogeneous exposure definitions; mid-lactation Holsteins
Heat load → water intake (dairy cattle)	[7]	Meta-analysis	Strong	Substantial between-study heterogeneity; cooling water rarely reported separately
Heat stress → enteric CH_4_ yield and intensity	[11,12]	Single crossover trial; review	Limited	One trial, one breed
Heat stress → growth and mortality (pigs, field)	[27,28]	Single-farm observational study; review	Limited	Seasonal confounding; no control group
Heat stress → poultry performance and product quality	[34,35,37,56]	Reviews; systematic review	Moderate	Mostly controlled experiments
Heat stress → small-ruminant performance	[39,40,41,42]	Reviews; small experiments	Limited to moderate	Few dose-response data
Cooling energy → life cycle carbon emissions	[17]	Single modeling study	Limited	Two barn designs, one climate
Circular feed → lower LCA impact	[9,29,59]	Systematic reviews; one national LCA	Moderate; direction inconsistent	Allocation and substitution assumptions
Phytogenic additives → retained performance under heat stress	[30,36]	Review; single randomized field trial	Limited	No LCA of additive production
PLF/AI → net resource savings	[14,31,46,49,50]	Reviews; one predictive-model study	Limited	Footprint of digital infrastructure not assessed
Genetic heat tolerance → retained production	[13,18,23,24,48]	Systematic review; genomic evaluations	Moderate (cattle); limited (pigs, poultry)	Unfavorable genetic correlations with yield
Heat stress explicitly integrated into livestock LCA	—	None identified	Absent	Core gap addressed by this framework

Grading: strong, at least one meta-analysis or several consistent studies from more than one region; moderate, several primary studies or systematic reviews with some inconsistency; limited, single study, single farm or single region; absent, no direct study identified.

## Data Availability

The original contributions presented in this study are included in the article. Further inquiries can be directed to the corresponding author.

## Supplementary Materials

The following supporting information can be downloaded at https://www.mdpi.com/article/10.3390/ani16203290/s1: Supplementary File S1: HSCI calculator (Microsoft Excel workbook) reproducing the worked example (Table 6), the weight-sensitivity analysis and the pilot application to farm data (Table 7), with editable inputs, bounds and weights, together with the Monte Carlo uncertainty analysis of Equation (1), the robustness analysis of the pilot TR estimate and the sample-size calculation (Equation (5)).

## Abbreviations

The following abbreviations are used in this manuscript:

ADG	Average daily gain
AHP	Analytic Hierarchy Process
AI	Artificial intelligence
AWI	Animal welfare indicator score
BGHI	Black Globe-Humidity Index
CE	Circular economy
CH_4_	Methane
CO_2_	Carbon dioxide
CV	Cross-validation
DMI	Dry matter intake
ECM	Energy-corrected milk
EE	Environmental efficiency (HSCI component)
ERA5-Land	Fifth-generation ECMWF atmospheric reanalysis of the global climate, land component
FAO	Food and Agriculture Organization of the United Nations
GHG	Greenhouse gas
HLI	Heat Load Index
HS	Heat stress
HSCI	Heat-Stress Circularity Index
ICT	Information and communication technology
LCA	Life cycle assessment
LCC	Life cycle costing
N_2_O	Nitrous oxide
NH_3_	Ammonia
PCA	Principal component analysis
PLF	Precision livestock farming
PR	Productivity retention (HSCI component)
PRISMA	Preferred Reporting Items for Systematic Reviews and Meta-Analyses
PV	Photovoltaic
RC	Resource circularity (HSCI component)
RFID	Radio-frequency identification
THI	Temperature–humidity index
TR	Thermal resilience (HSCI component)
US	United States
W	Welfare (HSCI component)
